# Clinical Risk and Outpatient Therapy Utilization for COVID-19 in the Medicare Population

**DOI:** 10.1001/jamahealthforum.2023.5044

**Published:** 2024-01-26

**Authors:** Andrew D. Wilcock, Stephen Kissler, Ateev Mehrotra, Brian E. McGarry, Benjamin D. Sommers, David C. Grabowski, Yonatan H. Grad, Michael L. Barnett

**Affiliations:** 1 Harvard Medical School, Boston, Massachusetts; 2 Beth Israel Deaconess Medical Center, Boston, Massachusetts; 3 University of Rochester Medical Center, Rochester, New York; 4 Harvard T.H. Chan School of Public Health, Boston, Massachusetts; 5 Division of General Internal Medicine and Primary Care, Department of Medicine, Brigham and Women’s Hospital, Boston, Massachusetts

## Abstract

**Importance:**

Multiple therapies are available for outpatient treatment of COVID-19 that are highly effective at preventing hospitalization and mortality. Although racial and socioeconomic disparities in use of these therapies have been documented, limited evidence exists on what factors explain differences in use and the potential public health relevance of these differences.

**Objective:**

To assess COVID-19 outpatient treatment utilization in the Medicare population and simulate the potential outcome of allocating treatment according to patient risk for severe COVID-19.

**Design, Setting, and Participants:**

This cross-sectional study included patients enrolled in Medicare in 2022 across the US, identified with 100% Medicare fee-for-service claims.

**Main Outcomes and Measures:**

The primary outcome was any COVID-19 outpatient therapy utilization. Secondary outcomes included COVID-19 testing, ambulatory visits, and hospitalization. Differences in outcomes were estimated based on patient demographics, treatment contraindications, and a composite risk score for mortality after COVID-19 based on demographics and comorbidities. A simulation of reallocating COVID-19 treatment, particularly with nirmatrelvir, to those at high risk of severe disease was performed, and the potential COVID-19 hospitalizations and mortality outcomes were assessed.

**Results:**

In 2022, 6.0% of 20 026 910 beneficiaries received outpatient COVID-19 treatment, 40.5% of which had no associated COVID-19 diagnosis within 10 days. Patients with higher risk for severe disease received less outpatient treatment, such as 6.4% of those aged 65 to 69 years compared with 4.9% of those 90 years and older (adjusted odds ratio [aOR], 0.64 [95% CI, 0.62-0.65]) and 6.4% of White patients compared with 3.0% of Black patients (aOR, 0.56 [95% CI, 0.54-0.58]). In the highest COVID-19 severity risk quintile, 2.6% were hospitalized for COVID-19 and 4.9% received outpatient treatment, compared with 0.2% and 7.5% in the lowest quintile. These patterns were similar among patients with a documented COVID-19 diagnosis, those with no claims for vaccination, and patients who are insured with Medicare Advantage. Differences were not explained by variable COVID-19 testing, ambulatory visits, or treatment contraindications. Reallocation of 2022 outpatient COVID-19 treatment, particularly with nirmatrelvir, based on risk for severe COVID-19 would have averted 16 503 COVID-19 deaths (16.3%) in the sample.

**Conclusion:**

In this cross-sectional study, outpatient COVID-19 treatment was disproportionately accessed by beneficiaries at lower risk for severe infection, undermining its potential public health benefit. Undertreatment was not driven by lack of clinical access or treatment contraindications.

## Key Points

**Question:**

How did access to outpatient COVID-19 therapy vary across patients in the Medicare program in 2022, and what clinical factors explain these differences?

**Findings:**

In this cross-sectional study of patients enrolled in Medicare in 2022, those at the highest risk for severe COVID-19 infection received COVID-19 therapy less often than those with the least risk. Disparities in therapy access were found by patient age, race and ethnicity, Medicaid eligibility, and nursing home residence.

**Meaning:**

Outpatient COVID-19 treatment utilization was disproportionately more among beneficiaries at lower risk for severe infection, potentially undermining its public health benefit.

## Teaser

This cross-sectional study assesses utilization of COVID-19 outpatient treatment in the Medicare population and simulates the potential outcome of allocating treatment according to patient risk for severe COVID-19.

## Introduction

Multiple therapeutic agents have been developed for the treatment of outpatient COVID-19 infection that are highly effective at preventing hospitalization and mortality, particularly for those at high risk for severe complications.^1,2,3^ The first available outpatient COVID-19 treatments, monoclonal SARS-CoV-2 antibodies (mAbs), required access to an infusion center, likely explaining why fewer than 7% of Medicare beneficiaries with confirmed COVID-19 used mAbs in 2020 and 2021.^4,5^ In December 2021, emergency use authorization of the oral agents nirmatrelvir (available via prescription as a formulation with ritonavir to boost drug levels)^6^ and molnupiravir^7^ for outpatient COVID-19 treatment raised hopes that access would improve,^8^ especially because these treatments were provided at no cost to patients.^9,10^ This was followed by the approval of intravenous remdesivir, which had previously been limited to inpatient use, for outpatient therapy in January 2022.^11^

Early evidence suggests that use of outpatient oral treatment has been low. One study found that less than 13% of patients with COVID-19 used an oral antiviral throughout the first half of 2022,^9^ while another estimated that 38% of confirmed COVID-19 infections received oral treatment in late 2022.^10^ Wide geographic and demographic variation in use of nirmatrelvir was found. For instance, non-Hispanic White patients used nirmatrelvir 50% more frequently than Black or Hispanic patients,^9,12^ and use was higher in more advantaged neighborhoods.^13,14^ The mechanisms underlying low and disparate use of COVID-19 therapies are unclear and have important implications for public health and policies to promote health equity. Possible factors include differential access to clinicians willing to prescribe antivirals or administer antibodies, concerns about drug interactions, patient preferences, the timing of when patients sought care (eg, tested for COVID-19 only after going to the hospital), or numerous nonclinical barriers, such as poverty and structural racism.^15^

To fill this knowledge gap, we assessed use of outpatient COVID-19 therapies in the Medicare population in 2022, accounting for individual patient factors not reflected in the previous literature,^10,12,13,14^ such as access to outpatient care, potential contraindications to therapy use, clinical need on the basis of risk for COVID-19–related mortality, and physician variation in prescribing. We also simulated the potential outcomes of whether more optimal allocation of treatment may have averted COVID-19–related hospitalizations and deaths.

## Methods

### Data Sources and Study Populations

In this cross-sectional study, the primary data sources used were the Medicare Master Beneficiary Summary and Standard Analytic claim files, including 100% Part D prescription event files. We also used the Minimum Data Set 3.0 to capture nursing home utilization. The Office of Human Research Administration at Harvard T.H. Chan School of Public Health approved this study. This study followed the Strengthening the Reporting of Observational Studies in Epidemiology (STROBE) reporting guideline. Informed consent was not obtained because of sample size, and the data were deidentified.

The study period for the main analysis was the calendar year 2022. The study population included all Medicare beneficiaries continuously enrolled in traditional fee-for-service Medicare parts A, B and D from January through December 2022 or until beneficiary death. Due to the lack of claims data for Medicare Advantage enrollees in the Standard Analytic Files, beneficiaries concurrently enrolled in a Medicare Advantage plan at any point during 2022 were excluded from this study’s main analysis. We also excluded beneficiaries living outside of the 50 US states and Washington, DC.

In sensitivity analyses, we examined use of COVID-19 treatments in some groups of enrollees excluded from this study’s main analysis. Because Medicare Advantage enrollees are included in the Master Beneficiary Summary Files and in the 100% Part D prescription event files, we examined prescription-based COVID-19 therapy use in the sample of Medicare Advantage enrollees continuously enrolled in Parts A, B and D in 2022. We also relaxed the continuous enrollment requirement and examined total volumes of antiviral and antibody treatment across all fee-for-service Medicare beneficiaries regardless of length of enrollment during the period from 2020 to 2022.

### Study Outcomes

Use of any outpatient oral or intravenous (IV) COVID-19 therapy in 2022 was the primary outcome. This outcome was defined for all beneficiaries in the study population allowing us to capture all observed and unobserved COVID-19 infections regardless of varying test availability and reporting.^16^

Therapies were outpatient oral antivirals nirmatrelvir and molnupiravir, as well as IV antiviral remdesivir (given as an outpatient infusion) and outpatient IV monoclonal SARS-CoV-2 antibodies (bamlanivimab/etesevimab, casirivimab/imdevimab, sotrovimab, and bebtelovimab). We identified use of oral antivirals from the Part D event claims using National Drug Codes and identified the administration of IV antivirals and antibodies from the Carrier (professional Part B) and Outpatient claim files using *Current Procedural Terminology (CPT)*/Healthcare Common Procedure Coding System (HCPCS) codes (see eMethods 1 in Supplement 1 for listings of codes used for each therapy).

We evaluated several other COVID-19–related services as secondary outcomes. As a measure of COVID-19 burden across patients, we assessed the incidence of severe infections by flagging beneficiaries hospitalized with a primary diagnosis of COVID-19 (*International Statistical Classification of Diseases and Related Health Problems, Tenth Revision [ICD-10]* code: U07.1). This measure is now used by the Centers for Disease Control and Prevention instead of case counts to quantify community levels of COVID-19 incidence.^17,18^ We also identified whether a beneficiary had any diagnosis for COVID-19 in 2022 (inclusive of any primary or secondary diagnosis on any facility or professional claim). To assess whether patients also differed on other outpatient COVID-19–related services, we created indicators for whether the beneficiary had an ambulatory visit (ie, evaluation and management visit with a physician or advanced practice clinician, such as a nurse practitioner) where the primary diagnosis was COVID-19, and an indicator for any antibody/antigen testing for COVID-19 using codes^19^ (eMethods 1 in Supplement 1) recorded on any claim in 2022.

### Patient Characteristics

Demographics were obtained from Master Beneficiary Summary Files, including age (as of January 1, 2022), sex, urban or rural residence (using Rural-Urban Continuum Code^20^ for the beneficiary’s zip code), original Medicare entitlement reason (age, disability, or end-stage kidney disease), and dual eligibility for Medicaid during any month of 2022. For each beneficiary, we added indicators and counts of chronic conditions included in the Chronic Conditions Warehouse (CCW)^21^ as of December 31, 2020. To define race and ethnicity, we used the Research Triangle Institute variable in the Medicare Beneficiary Summary File, which identifies Black and Hispanic beneficiaries with high sensitivity and specificity, but there is underrecognition of other races.^22^ Self-reported race and ethnicity included the following categories: American Indian and Alaska Native, Asian and Pacific Islander, Black (or African-American), Hispanic, non-Hispanic White, other, and unknown. Because of their smaller size, we created an other race category, which accounted for 3.7% of this study’s sample and included individuals with American Indian or Alaska Native, unknown, or other race and ethnicity.

Using skilled nursing facility records from the Minimum Data Set, version 3.0 (Centers for Medicare & Medicaid Services), we added an indicator for whether the beneficiary had any short-term or long-term stay in a nursing home during 2022.^23^ Because treatment utilization could depend on ability to access care remotely, we additionally captured whether beneficiaries received any outpatient telemedicine service in 2022, using previously published definitions.^24,25,26^ For each beneficiary, we identified whether they had a Medicare claim for COVID-19 vaccination (eMethods 1 in Supplement 1) from January 2020 to December 2022, classified as having at least 1 vaccine claim or having no vaccine claims, which likely includes both beneficiaries who were not vaccinated and those who received vaccines without a Medicare claim.

We also created a composite score for COVID-19 mortality risk (or COVID-19 severity risk) for each person in this sample. This score serves as a measure of the aggregate observable risk across demographic and comorbidities that a clinician may consider in deciding to prescribe COVID-19 therapy. Scores were based on estimated coefficients from a linear regression model of mortality within 21 days of a COVID-19 diagnosis using data from 2021 (ie, prior to the study period). We then generated a score for each beneficiary using 2022 characteristics (eMethods 2 in Supplement 1 for specification details and eTable 1 in Supplement 1 for model estimates). We assigned beneficiaries to quintiles of COVID-19 severity risk. We attributed beneficiaries to primary care practices using previously published methods^27^ based on the plurality of where beneficiaries receive primary care (eMethods 3 in Supplement 1).

### Contraindications to Nirmatrelvir

Nirmatrelvir has multiple potential contraindications, including specific clinical conditions (kidney or liver disease) or drug-drug interactions which could be a major impediment to its use.^28^ Other COVID-19 therapies do not have such complex and commonly occurring contraindications. Using treatment guidelines,^29^ we identified 2 tiers of drug safety risk for nirmatrelvir: tier 1 drugs, which need to be closely monitored or those that require dosage adjustment, and tier 2 drugs, which are contraindicated and must be held if nirmatrelvir is prescribed. For each beneficiary, interactions were identified by filled prescriptions from October 2021 to December 2022 (eMethods 3 in Supplement 1). We created 3 levels of potential nirmatrelvir contraindications: (1) no liver or kidney disease and no drug-drug interactions (no contraindication), (2) any tier 1 drug-drug interactions or liver/kidney disease (moderate contraindication), and (3) any tier 2 drug interaction (severe contraindication).

### Statistical Analysis

First, we described the percentage of beneficiaries who received outpatient COVID-19 treatment or were hospitalized due to COVID-19, overall and by patient characteristic, and estimated adjusted odds ratios (aORs) and 95% CIs using multivariable logistic regressions, adjusting for all factors described previously, with robust standard errors clustered at the hospital referral region (HRR) to account for potential nonindependence of observations due to geographic treatment patterns. We examined any COVID-19 therapy, as well as any oral therapy and any IV therapy in separate models.

Second, we examined the role of geography or primary care practice. Some of the observed differences in outpatient treatment use across demographic groups could be driven by variation across geographic regions in use of treatment or variation across primary care practices in their use of treatment. We quantified the importance of HRR or attributed primary care practice by assessing how much additional variation in therapy utilization was explained in models with either HRR or primary care practice fixed effects, and whether the baseline patient characteristic coefficients were changed after inclusion of the fixed effects.

We replicated the analyses in several special populations. First, we evaluated COVID-19 therapy use among beneficiaries with a COVID-19 diagnosis in 2022. Given the large fraction of therapies prescribed without a diagnosis, we also documented the percentage of therapies with and without a proximate COVID-19 diagnosis and differences in patient characteristics between therapies administered with and without a diagnosis.

Next, we examined oral prescription outcomes for Medicare Advantage enrollees with a more limited set of covariates available in enrollment and Minimum Data Set files (age, sex, race and ethnicity, Medicaid eligibility, geography, and nursing home/community residence) because the Part D file contains full information on their prescription coverage. We compared these results with those for the fee-for-service population using the covariates available for both populations. In addition, because most randomized trial data on nirmatrelvir are observed in an unvaccinated population, we replicated selected analyses for just the fee-for-service population with no observable vaccination claims. We also evaluated whether excluding beneficiaries younger than 65 years, who largely qualify for Medicare through disability, affected this study’s estimates.

Finally, we described outcomes using the COVID-19 severity risk quintiles identified in this study, and we conducted a simulation to quantify the potential outcome of a counterfactual scenario of nirmatrelvir distribution based on clinical risk on the number of adverse events in 2022, including hospitalizations and death (eMethods 4 in Supplement 1). We focused on nirmatrelvir alone because it comprised most therapies in 2022, and the real-world effectiveness of molnupiravir is uncertain.^30^ Specifically, we modeled the scenario where the same number of nirmatrelvir doses were provided in 2022 but reallocated in proportion to each COVID-19 severity risk quintile’s contribution to overall mortality (eg, if the top quintile group comprised 50% of COVID-19 deaths, they would receive 50% of treatment). Given high rates of home diagnosis that may not result in a health care encounter,^16^ we assumed that 60% of all COVID-19 infections were observed within Medicare claims and also examined results assuming 50% or 75%. We assumed a 40% reduction in hospitalization and 70% reduction in death associated with nirmatrelvir use, within the range established by observational evidence in contemporaneous, vaccinated populations.^30,31,32,33,34^ To assess uncertainty in the estimate of the reduction in mortality attainable by redistributing treatment according to clinical severity risk, we considered a lower plausible bound of 50% case ascertainment and 50% treatment efficacy, and an upper bound of 75% case ascertainment and 90% treatment efficacy. We conducted all analyses in Stata, version 17.0 (StataCorp), and R statistical software, version 3.5.0 (The R Project for Statistical Computing).

## Results

**Figure 1.**
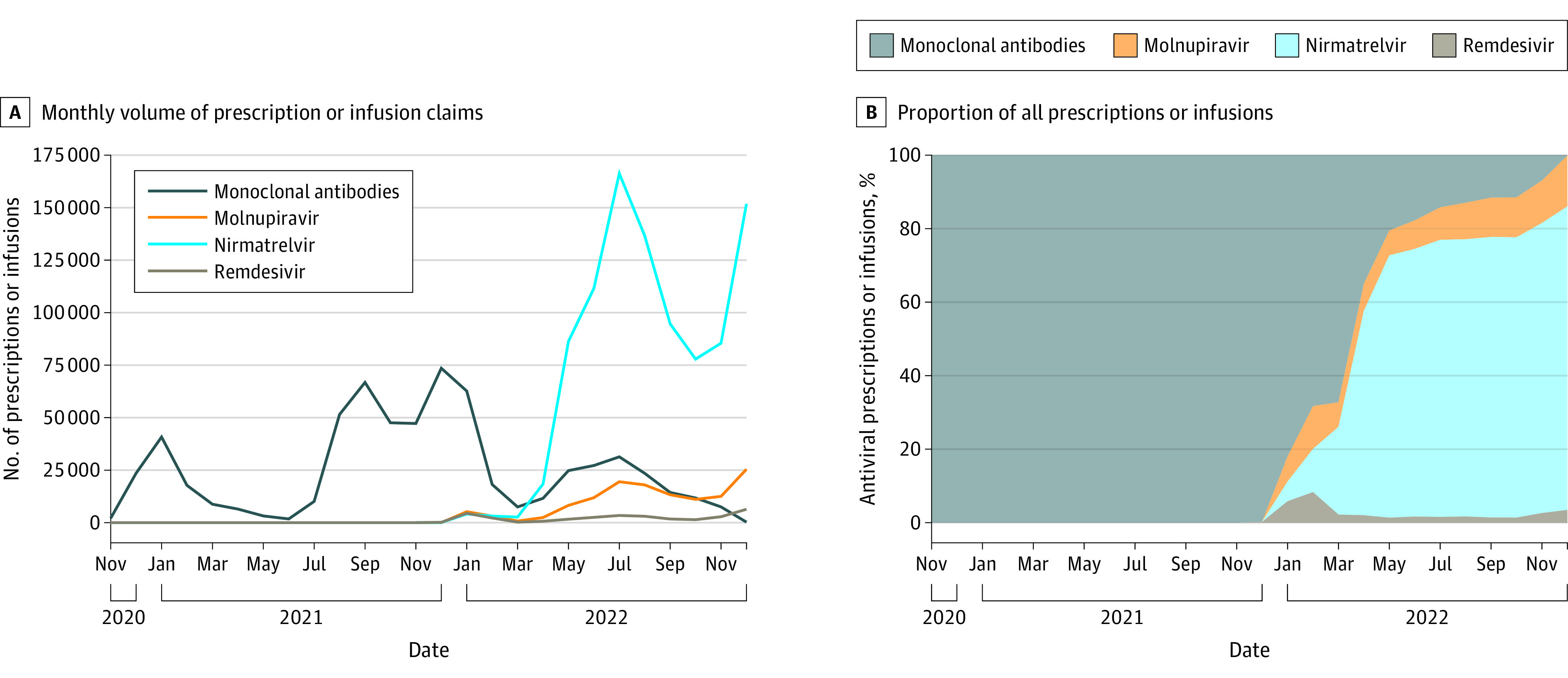
Monthly Counts and Proportion of Infusions or Prescriptions of Outpatient COVID-19 Therapies Delivered to Fee-for-Service Medicare A, The left panel shows the monthly volume of prescription claims (for oral medications) or infusion claims (for intravenous medications) of outpatient COVID-19 therapies from November 2020 to December 2022 for all Medicare beneficiaries, regardless of enrollment. The main analytic sample in subsequent exhibits focuses on continuously enrolled beneficiaries in 2022 alone. B, the graph shows the proportion of all prescriptions or infusions in a given month in 1 of 4 categories (monoclonal antibodies, molnupiravir, nirmatrelvir, or remdesivir). Prior to late 2021, only monoclonal antibodies were available for outpatient treatment of COVID-19.

From 2020 to 2022, there were 1 742 100 outpatient treatments observed in claims for 1 665 166 beneficiaries across the entire Medicare fee-for-service population (Figure 1). Before December 2021, therapies were limited to IV monoclonal antibodies; nirmatrelvir and molnupiravir became the dominant therapies in 2022.

The main analytic sample included 20 026 910 beneficiaries in fee-for-service Medicare continuously enrolled in parts A, B and D in 2022 (or until death in 2022), regardless of receipt of COVID-19 therapy. In this sample, 6.0% of beneficiaries received any COVID-19 therapy (Table 1). Among those with a COVID-19 diagnosis in 2022, 23.0% received therapy within 10 days before or after the diagnosis (eTable 2 in Supplement 1), and among those receiving any therapy, 40.5% of oral prescriptions, and 1.5% of IV doses had no COVID-19 diagnosis claim within 10 days of treatment (eTable 3 in Supplement 1), with heterogeneity across patients. For example, White patients were more likely to have treatment without an associated COVID-19 diagnosis compared with Black patients (40.7% and 32.0%, respectively; eTable 4 in Supplement 1).

**Table 1.** Unadjusted Rates of Outpatient COVID-19 Therapy by Demographics in 2022, Among Those With or Without a COVID-19 Diagnosis

Characteristic	Beneficiaries, %	%			
		COVID-19 therapy			COVID-19 admission
		Any	Oral	IV	
Total, No.	20 026 910	6.0	5.1	1.0	1.0
Age, y					
<40	2.5	2.2	1.7	0.5	0.5
40-49	2.3	3.1	2.3	0.8	0.8
50-59	3.9	3.5	2.8	0.8	1.0
60-64	3.2	4.1	3.2	0.9	1.2
65-69	25.2	6.4	5.6	0.8	0.5
70-74	24.1	6.8	5.9	1.0	0.6
75-79	17.1	6.7	5.6	1.1	1.0
80-84	11.0	6.2	5.0	1.2	1.5
85-89	6.3	5.7	4.5	1.2	2.0
≥90	4.3	4.9	3.8	1.1	2.6
Sex					
Female	56.8	6.1	5.2	1.0	0.9
Male	43.2	6.0	5.0	1.0	1.0
Race					
Asian	3.1	6.2	5.7	0.6	0.8
Black	6.5	3.0	2.5	0.6	1.3
Hispanic	5.2	4.3	3.6	0.7	1.2
White	81.5	6.4	5.4	1.0	0.9
Other race^a^	3.7	6.8	5.9	1.0	0.7
Geography					
Urban	76.7	6.3	5.4	1.0	1.0
Rural	23.3	5.2	4.2	1.1	0.9
Medicaid eligibility					
No	78.0	6.8	5.8	1.0	0.8
Yes	22.0	3.3	2.6	0.7	1.5
Original reason for Medicare eligibility					
Age	80.2	6.5	5.6	1.0	0.9
Disability	19.2	4.0	3.1	0.9	1.2
ESKD	0.6	5.5	2.0	3.7	4.6
Institution					
Community	93.6	6.2	5.3	1.0	0.7
Nursing home	6.4	4.2	3.0	1.2	4.9
Chronic condition count^b^					
No information	4.9	5.7	5.0	0.7	0.4
0	12.8	5.0	4.5	0.6	0.3
1-3	19.0	5.5	4.9	0.6	0.3
4-5	17.7	6.4	5.6	0.8	0.5
6-9	30.7	6.6	5.5	1.2	0.9
≥10	19.8	6.1	4.6	1.6	2.5
Vaccination claims^c^					
No claim	26.0	4.0	3.1	1.0	1.5
≥1 claim	74.0	6.7	5.8	1.0	0.8
Nirmatrelvir contraindication^d^					
None	16.5	4.3	3.9	0.4	0.2
Moderate	72.4	6.4	5.5	1.0	1.0
Severe	11.2	6.1	4.5	1.7	1.9
Telemedicine^e^					
No use	65.0	5.5	4.7	0.8	0.8
Use	35.0	7.0	5.9	1.2	1.2

Abbreviations: ESKD, end-stage kidney disease; IV, intravenous.

^a^ Other race includes beneficiaries with American Indian or Alaska Native; unknown; or other race and ethnicity. These are the 3 options for self-identified race in the Medicare enrollment file besides Asian and Pacific Islander, Black, Hispanic, and non-Hispanic White. Less than 1% of beneficiaries are coded as unknown race.

^b^ For the chronic condition count, the presence of 27 conditions was gathered from the Chronic Condition Data Warehouse, which uses claims since 1999 to describe Medicare beneficiaries’ accumulated chronic disease burden. Comorbidities were defined as any condition present as of December 31, 2020.

^c^ Receipt of vaccination for COVID-19 over January 2020 through December 2022, classified as having ≥1 vaccine claims or having no vaccine claims, which could includes both unvaccinated beneficiaries and those who received vaccines without billing Medicare.

^d^ We combined drug tiers and indicators for kidney or liver disease into 3 levels of potential nirmatrelvir contraindications: (1) no liver or kidney disease and no drug-drug interactions (no contraindication), (2) any tier 1 interactions and/or liver/kidney disease (moderate contraindication), and (3) any tier 2 drug interaction, with or without liver/kidney disease (severe contraindication).

^e^ Telemedicine visits were identified through modifiers GT, GQ, or 95 on eligible outpatient services or *Current Procedural Terminology *codes 99441-99443.

### Differences in COVID-19 Therapy Use in 2022

**Figure 2.**
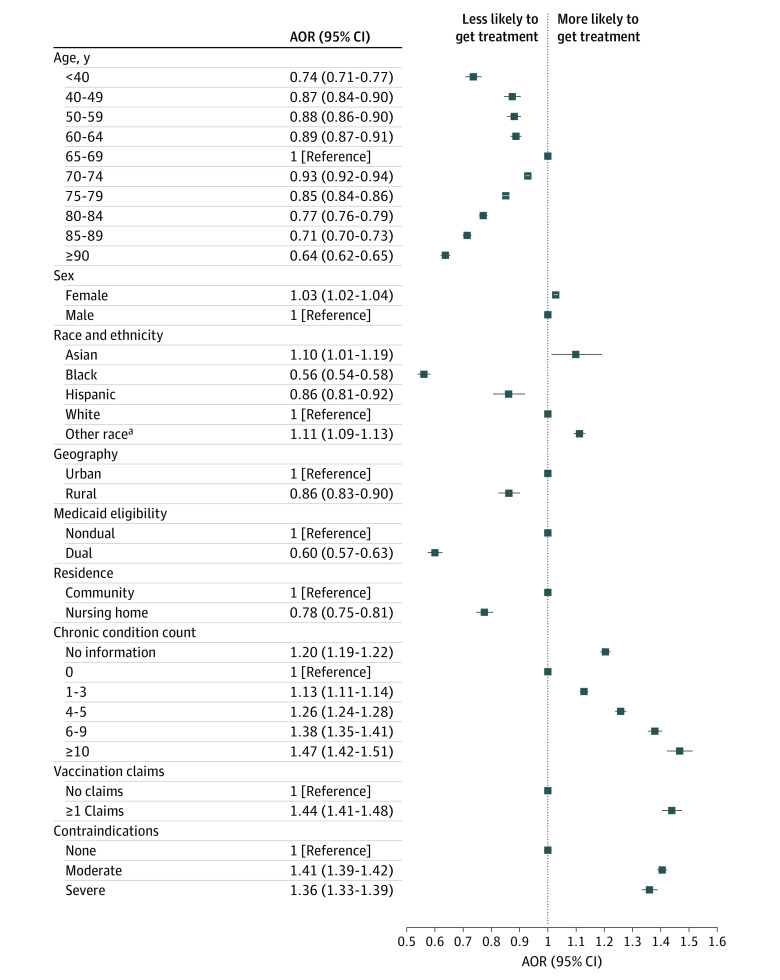
Adjusted Odds of Receiving Any Outpatient COVID-19 Treatment by Selected Patient Characteristics Forest plot showing adjusted odds ratios (aORs) for a beneficiary-level logistic regression model with the outcome of receipt of any COVID-19 treatment in 2022, adjusting for all characteristics shown in Table 1 with robust standard errors clustered at the hospital referral region. Characteristics not shown for visualization: original reason for Medicare eligibility and telemedicine use. ^a^Other race includes beneficiaries with American Indian or Alaska Native; unknown; or other race and ethnicity. These are the 3 options for self-identified race in the Medicare enrollment file besides Asian and Pacific Islander, Black, Hispanic, and non-Hispanic White. Less than 1% of beneficiaries are coded as unknown race.

Use of outpatient COVID-19 treatment was generally lower among patients at higher risk for severe COVID-19 infection, as assessed by COVID-19 hospitalization rate (Table 1). For example, treatment rates were 4.9% among those 90 years and older and 6.4% for those aged 65 to 69 years (aOR, 0.64 [95% CI, 0.62-0.65]). Among those with nursing home use in 2022, treatment rates were 4.2%, compared with 6.2% among community-dwelling residents (aOR, 0.78 [95% CI, 0.75-0.81]) (Figure 2; eTable 5 in Supplement 1). Substantial differences by race and ethnicity and rural/urban residence were found. For Black patients, 3.0% received COVID-19 therapy, compared with 6.4% of White patients (aOR, 0.56 [95% CI, 0.54-0.58]). For Hispanic patients, 4.3% received COVID-19 therapy (aOR compared with White patients, 0.86 [95% CI, 0.81-0.92]). Rural beneficiaries were also less likely to receive treatment than urban residents (5.2% and 6.3%, respectively; aOR, 0.86 [95% CI, 0.83-0.90]). Unadjusted treatment rates were lower for those with 10 or more chronic conditions compared with 6 to 9 (6.1% and 6.6%, respectively), though adjusted odds of treatment increased monotonically with more chronic conditions. Beneficiaries with any telemedicine use in 2022 also received treatment more frequently than those who did not (7.5% and 5.5%, respectively). These patterns were similar among patients with a COVID-19 diagnosis in 2022, the 26% of patients with no claims for vaccination, those 65 years and older, and patients insured with Medicare Advantage who used oral therapy (eTables 2, 6-8 in Supplement 1).

**Figure 3.**
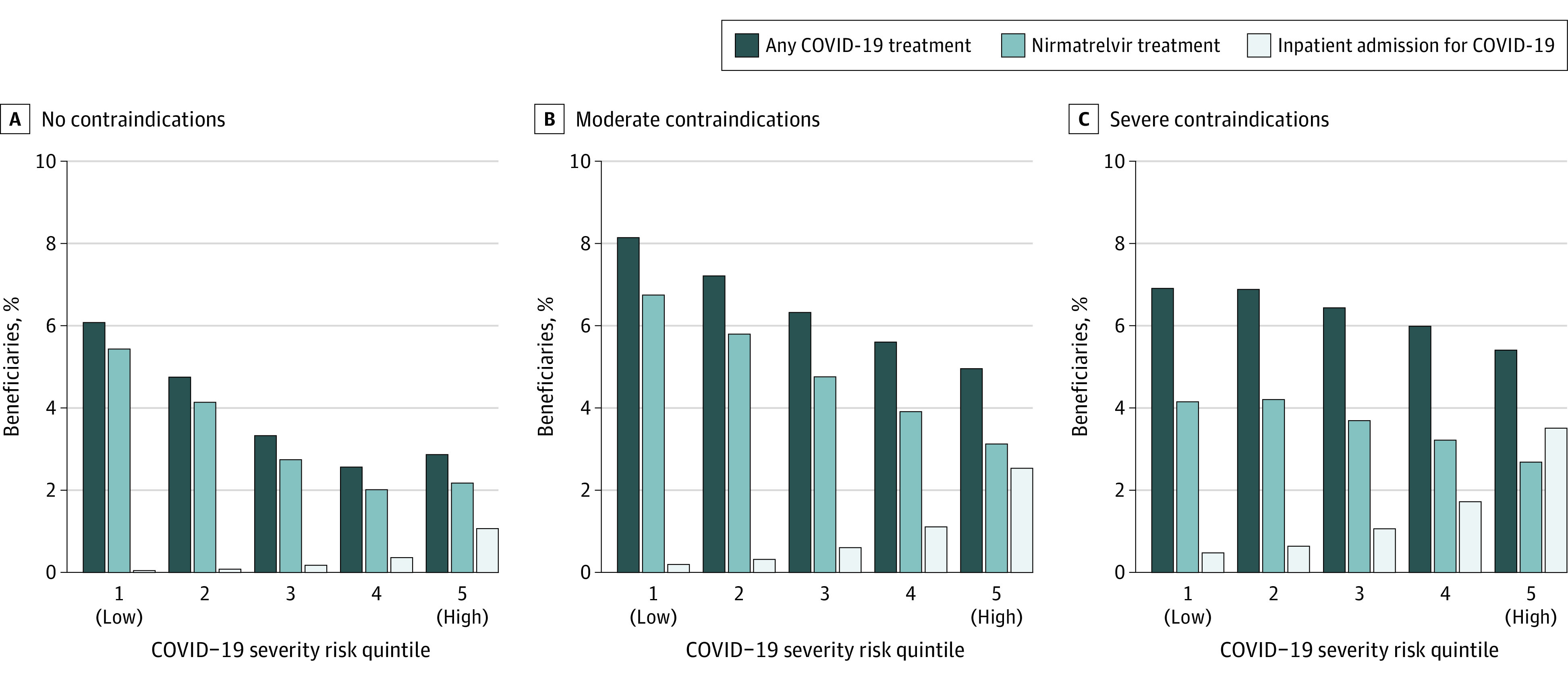
Rates of Outpatient COVID-19 Therapy and Inpatient Admissions by COVID-19 Severity Risk and Nirmatrelvir Contraindications The figure shows rates of any outpatient COVID-19 treatment, nirmatrelvir treatment, and inpatient admission for Medicare beneficiaries in 2022 stratified by their COVID-19 severity risk quintile. This score serves as a measure of the aggregate observable risk across dozens of characteristics that a clinician may consider in deciding to prescribe COVID-19 therapy. Scores were based on estimated coefficients from a linear regression model of mortality following COVID-19 (within 21 days) in 2021, using each beneficiary’s characteristics in 2022 (eMethods 2 in Supplement 1). Beneficiaries were assigned to COVID-19 severity risk quintiles.

An inverse association was found between patients’ overall COVID-19 severity risk, which incorporates risk across all patient characteristics in Table 1, and receipt of outpatient COVID-19 treatment (Figure 3; eTable 9 in Supplement 1). Among patients in the highest risk quintile, in which 2.6% were hospitalized for COVID-19 in 2022, 4.9% received any treatment for COVID-19. Among patients in the lowest risk quintile, in which 0.2% were hospitalized, 7.5% received any treatment.

### Clinical Factors Potentially Associated With Differences in Outpatient Therapy Use

#### Contraindications to Nirmatrelvir

The presence of contraindications to nirmatrelvir (either drug-drug interactions with nirmatrelvir/ritonavir or kidney and/or liver disease) was associated with higher, not lower, rates of outpatient COVID-19 treatment. Beneficiaries with contraindications had higher baseline levels of both oral and IV treatment for COVID-19 (4.5% and 1.7% for severe contraindications; 5.5% and 1.0% for moderate contraindications) than those without contraindications (3.9% and 0.4%, Table 1). Among patients with no contraindication, nirmatrelvir use fell with increasing COVID severity risk (Figure 3).

#### Testing and Ambulatory Visits for COVID-19

Groups who less often received outpatient treatment did not test or attend ambulatory visits for COVID-19 less often (Table 2; eTable 9 in Supplement 1). For example, low-income patients (as captured by Medicaid dual eligibility) were less likely to receive any COVID-19 treatment compared with patients without dual eligibility (3.3% and 6.8%, respectively) but were more likely to have an ambulatory visit for COVID-19 (11.7% and 10.4%, respectively) or a testing claim in 2022 (35.0% and 32.2%, respectively). Similarly, people in a nursing home were less likely to receive any COVID-19 treatment compared with community-dwelling people, but more likely to have a COVID-19 visit (25.2% and 9.7%, respectively) or testing (52.5% and 31.5%, respectively) in 2022.

**Table 2.** Rates of Other Outpatient COVID-19 Services by Demographics in 2022

Characteristic	%		
	Beneficiaries	COVID-19 test	COVID-19 ambulatory visit
Total, No.	20 026 910	32.9	10.7
Age, y			
<40	2.5	32.0	8.6
40-49	2.3	34.6	10.0
50-59	3.9	35.8	10.4
60-64	3.2	35.3	10.6
65-69	25.2	31.3	9.5
70-74	24.1	32.7	10.3
75-79	17.1	33.8	11.2
80-84	11.0	33.7	12.0
85-89	6.3	33.2	12.9
≥90	4.3	32.8	13.7
Sex			
Female	56.8	33.8	10.9
Male	43.2	31.7	10.5
Race and ethnicity			
Asian	3.1	30.4	9.3
Black	6.5	33.8	10.5
Hispanic	5.2	34.3	11.2
White	81.5	32.9	10.8
Other^a^	3.7	32.8	10.2
Geography			
Urban	76.7	33.2	11.0
Rural	23.3	32.0	9.8
Medicaid eligibility			
No	78.0	32.3	10.4
Yes	22.0	35.1	11.9
Original reason for Medicare eligibility			
Age	80.2	32.1	10.6
Disability	19.2	35.7	11.0
ESKD	0.6	51.9	18.8
Institution			
Community	93.6	31.6	9.7
Nursing home	6.4	52.6	25.2
Chronic condition count^b^			
No information	4.9	30.1	8.9
0	12.8	26.1	7.7
1-3	19.0	27.1	8.1
4-5	17.7	30.6	9.5
6-9	30.7	35.1	11.3
≥10	19.8	41.7	15.3
Vaccination claims^c^			
No claim	26.0	28.7	10.8
≥1 claim	74.0	34.4	10.7
Nirmatrelvir contraindication^d^			
None	16.5	20.3	6.6
Moderate	72.4	34.3	11.1
Severe	11.2	42.5	14.1
Telemedicine^e^			
No use	65.0	30.0	9.3
Use	35.0	38.4	13.4

Abbreviation: ESKD, end-stage kidney disease.

^a^ Other race includes beneficiaries with American Indian or Alaska Native; unknown; or other race and ethnicity. These are the 3 options for self-identified race in the Medicare enrollment file besides Asian and Pacific Islander, Black, Hispanic, and non-Hispanic White. Less than 1% of beneficiaries are coded as unknown race.

^b^ For the chronic condition count, the presence of 27 conditions was gathered from the Chronic Condition Data Warehouse, which uses claims since 1999 to describe Medicare beneficiaries’ accumulated chronic disease burden. Comorbidities were defined as any condition present by as of December 31, 2020.

^c^ Receipt of vaccination for COVID-19 from January 2020 to December 2022, classified as having at least 1 vaccine claim or having no vaccine claims, which could include both unvaccinated beneficiaries and those who received vaccines without billing Medicare.

^d^ Drug tiers were combined with indicators for kidney or liver disease into 3 levels of potential nirmatrelvir contraindications: (1) no liver or kidney disease and no drug-drug interactions (no contraindication), (2) any tier 1 interactions and/or liver or kidney disease (moderate contraindication), and (3) any tier 2 drug interaction, with or without live or/kidney disease (severe contraindication).

^e^ Telemedicine visits were identified through modifiers GT, GQ, or 95 on eligible outpatient services or *Current Procedural Terminology* codes 99441-99443.

#### Geographic and Practice-Level Variation

Adjusting for HRRs did little to attenuate differences in outpatient COVID-19 treatment across patient characteristics (eTable 10 in Supplement 1) and did not explain a substantial fraction of the variation observed. Similarly, controlling for primary care practices explained little additional variation in therapy use, though the magnitude of some associations with COVID-19 treatment decreased modestly (eg, the adjusted coefficient for Black patients compared with White patients went from −2.25% to −2.04%).

### Simulated Reallocation of Nirmatrelvir

We simulated the potential differences in hospitalizations and mortality in the study sample if the nirmatrelvir doses delivered in 2022 had been reallocated in proportion to beneficiary risk for severe COVID-19 (eMethods 4 in Supplement 1). Only this therapy was focused on because it was the dominant therapy by the end of 2022. Assuming that 60% of all COVID-19 infections in the study sample had a diagnosis in claims, reallocation of nirmatrelvir according to patient risk would have averted 10 297 hospitalizations (5.4% relative reduction for the sample) and 16 503 deaths (16.3% reduction) in 2022, almost entirely due to the benefit in the highest risk group (eFigure in Supplement 1). As a lower bound, assuming 50% ascertainment and 50% treatment efficacy, reallocation of nirmatrelvir would have averted an estimated 9671 deaths (9.74% reduction); and as an upper bound, assuming 75% ascertainment and 90% treatment efficacy, reallocation would have averted an estimated 27 135 deaths (27.3% reduction).

## Discussion

This cross-sectional study assesses COVID-19 outpatient treatment utilization in the Medicare population and simulates the potential outcome of allocating treatment according to patient risk for severe COVID-19. Among Medicare beneficiaries in 2022, outpatient antiviral treatment was disproportionately accessed by individuals at lower risk for severe infection as measured by COVID-19 hospitalizations, a pattern of care that significantly limits the potential benefit of this treatment and may have contributed to thousands of avoidable deaths in the Medicare population. This misallocation of outpatient antiviral therapy contributes to well-recognized disparities in COVID-19 treatment for historically underserved groups like Black and Hispanic beneficiaries, patients dually eligible for Medicare and Medicaid, and rural residents.^4,5,9,10,12,13,14,35^ Moreover, we observed these disparities during a period in which antiviral and antibody therapy was free for patients. As treatment moves to an insurance-based model of reimbursement after the expiration of the Public Health Emergency, cost sharing may exacerbate disparities in treatment.^36^

The results of the present study provide novel evidence that these disparities are not associated with observable measures of outpatient treatment access (for example, COVID-19 testing and ambulatory visits), rates of contraindication to nirmatrelvir, and geographic or practice-level variation. Ambulatory visit and testing rates would need to vary substantially to explain these results, but we observed similar or higher COVID-19–related utilization in groups with less treatment. These findings were also similar among patients with a COVID-19 diagnosis and those with Medicare Advantage coverage. Given these treatments were free in 2022, cost barriers also cannot explain the findings.

Higher therapy use among telemedicine users and more frequent treatment without an associated diagnosis among White beneficiaries suggests that the ability to reach a prescriber outside of a standard ambulatory visit may play a role in observed inequities in access. It may be more difficult for lower-income, older, and rural beneficiaries to navigate the process of obtaining a COVID-19 therapy outside a face-to-face visit, a cascade including obtaining a home test, being aware of the benefits of outpatient treatment, contacting the clinician, and setting up a phone call or video telemedicine visit with a prescription written and filled. Taken together, these findings suggest that undertreatment of marginalized populations could be related to beneficiary preferences (eg, lack of belief in benefit, concern about adverse effects), differences in the timing of when patients sought care, structural barriers (eg, ability to communicate with clinician easily), bias in the likelihood of clinicians to provide treatment among those with infection (eg, prescribing nirmatrelvir if asked but not suggesting it to patients), or a combination of these factors.

### Limitations

First, we focus on the fee-for-service Medicare population, and these results may not generalize to other relevant populations. However, this population is critical to evaluate given that older adults had the highest morbidity and mortality during the pandemic. Second, this is an observational study; therefore, these results should be interpreted as exploratory associations, not causal relationships. Third, we cannot observe all incident COVID-19 infections in claims data. We addressed this bias by using a denominator of all enrolled beneficiaries, rather than limiting to those with a COVID-19 diagnosis, and examining COVID-19 hospitalizations as a proxy for COVID-19 disease burden following the approach used by the Centers for Disease Control and Prevention.^18^ Still, examining patterns of treatment among those with a COVID-19 diagnosis mirrored the results in the overall population. Fourth, we may not be capturing all COVID-19 treatment doses, because free doses may have been distributed without submitting insurance claims from community organizations. Finally, the simulation analysis uses multiple assumptions about outpatient treatment effectiveness and COVID-19 infection ascertainment which may be oversimplified. Given these limitations, the projections described in this study should be interpreted as a broad approximation of the potential magnitude of change with reallocation of therapy.

## Conclusions

In this cross-sectional study, we find a paradoxical association of lower outpatient COVID-19 treatment use among patients at highest risk for COVID-19 hospitalization and mortality: Black and Hispanic patients, dually eligible beneficiaries, adults 80 years and older, and beneficiaries using nursing homes. These results suggest that possible factors behind these disparities include some combination of patient preferences and patient-clinician factors, such as differences in willingness of clinicians to prescribe antivirals, rather than disparities in ability to access any clinician or any care. More clinically equitable distribution of outpatient treatment, particularly targeting those at highest risk for severe COVID-19, likely could have prevented thousands of hospitalizations and deaths. The misallocation of treatment existed despite no-cost availability of antiviral or antibody therapy and does not appear to be driven by testing, differences in accessing in-person care, or prevalence of contraindications. Correcting this disparity will require individual practices and health systems to interrogate their patterns of care and identify barriers to access for patients most at risk for adverse outcomes due to COVID-19 infection.

## Notes

Supplement 1. **eMethods 1.** Therapy, Testing, and Vaccination Codes **eMethods 2.** Model Specification **eMethods 3.** Primary Care Practice Attribution and Nirmatrelvir Contraindications **eMethods 4.** Details on Simulated Reallocation of Nirmatrelvir **eTable 1.** Mortality Following COVID-19 Diagnosis Risk Model Coefficients **eTable 2.** Rates of COVID-19 Therapy by demographics Among Patients With a COVID-19 Diagnosis in 2022 **eTable 3.** Proportion of Treatment Doses Proximate to a COVID-19 Diagnosis **eTable 4.** Characteristics of Oral Therapy Use With and Without COVID-19 Diagnosis **eTable 5.** Rates of COVID-19 Therapy by Demographics in 2022 **eTable 6.** Rates of COVID-19 Therapy by Demographics Among the Unvaccinated in 2022 **eTable 7.** Rates of COVID-19 Therapy by Demographics Among the Aged 65 Years and Over in 2022 **eTable 8.** Rates of Oral COVID-19 Therapy by Basic Demographics Among Medicare Advantage and Traditional Medicare in 2022 **eTable 9.** Rates of COVID-19 Therapies and Other Services by Severity Risk Quintile **eTable 10.** Impact of Further Adjustment for Hospital Region or Primary Care Practice on Adjusted Effect Sizes **eFigure.** Hospitalizations and Mortality Prevent With Counterfactual Redistribution of Nirmatrelvir in 2022

Supplement 2.
Data Sharing Statement
