# Supplementary material for: Clinical Risk and Outpatient Therapy Utilization for COVID-19 in the Medicare Population
Source: JAMA Health Forum. 2024 Jan 26;5(1):e235044. doi: 10.1001/jamahealthforum.2023.5044 (PMC10818223; doi:10.1001/jamahealthforum.2023.5044)
Supplement: Supplement 1. — eMethods 1. Therapy, Testing, and Vaccination Codes eMethods 2. Model Specification eMethods 3. Primary Care Practice Attribution and Nirmatrelvir Contraindications eMethods 4. Details on Simulated Reallocation of Nirmatrelvir eTable 1. Mortality Following COVID-19 Diagnosis Risk Model Coefficients eTable 2. Rates of COVID-19 Therapy by demographics Among Patients With a COVID-19 Diagnosis in 2022 eTable 3. Proportion of Treatment Doses Proximate to a COVID-19 Diagnosis eTable 4. Characteristics of Oral Therapy Use With and Without COVID-19 Diagnosis eTable 5. Rates of COVID-19 Therapy by Demographics in 2022 eTable 6. Rates of COVID-19 Therapy by Demographics Among the Unvaccinated in 2022 eTable 7. Rates of COVID-19 Therapy by Demographics Among the Aged 65 Years and Over in 2022 eTable 8. Rates of Oral COVID-19 Therapy by Basic Demographics Among Medicare Advantage and Traditional Medicare in 2022 eTable 9. Rates of COVID-19 Therapies and Other Services by Severity Risk Quintile eTable 10. Impact of Further Adjustment for Hospital Region or Primary Care Practice on Adjusted Effect Sizes eFigure. Hospitalizations and Mortality Prevent With Counterfactual Redistribution of Nirmatrelvir in 2022 [file jamahealthforum-e235044-s001.pdf]

Supplemental Online Content

Wilcock AD, Kissler S, Mehrotra A, et al. Clinical risk and outpatient therapy utilization for COVID-19 in the Medicare population. *JAMA Health Forum.* 2024;5(1):e235044. doi:10.1001/jamahealthforum.2023.5044

eMethods 1. Therapy, Testing, and Vaccination Codes

eMethods 2. Model Specification

eMethods 3. Primary Care Practice Attribution and Nirmatrelvir Contraindications

eMethods 4. Details on Simulated Reallocation of Nirmatrelvir

eTable 1. Mortality Following COVID-19 Diagnosis Risk Model Coefficients

eTable 2. Rates of COVID-19 Therapy by demographics Among Patients With a COVID-19 Diagnosis in 2022

eTable 3. Proportion of Treatment Doses Proximate to a COVID-19 Diagnosis

eTable 4. Characteristics of Oral Therapy Use With and Without COVID-19 Diagnosis

eTable 5. Rates of COVID-19 Therapy by Demographics in 2022

eTable 6. Rates of COVID-19 Therapy by Demographics Among the Unvaccinated in 2022

eTable 7. Rates of COVID-19 Therapy by Demographics Among the Aged 65 Years and Over in 2022

eTable 8. Rates of Oral COVID-19 Therapy by Basic Demographics Among Medicare Advantage and Traditional Medicare in 2022

eTable 9. Rates of COVID-19 Therapies and Other Services by Severity Risk Quintile

eTable 10. Impact of Further Adjustment for Hospital Region or Primary Care Practice on Adjusted Effect Sizes

eFigure. Hospitalizations and Mortality Prevent With Counterfactual Redistribution of Nirmatrelvir in 2022

This supplemental material has been provided by the authors to give readers additional information about their work.

© 2024 Wilcock AD et al. *JAMA Health Forum.*

eMethods 1. Therapy, testing, and vaccination codes

eMethods Table A. NDC codes used to identify oral COVID-19 therapies nirmatrelvir and molnupiravir in the part D event files

NDC	Name	Package Description	Claims (n) in 2022
00069108530	nirmatrelvir and ritonavir	5 blister pack in 1 carton	1,273,292
00069034530	nirmatrelvir and ritonavir	5 blister pack in 1 carton	427,532
00069110120	nirmatrelvir and ritonavir	5 blister pack in 1 carton	187,102
00069108506	nirmatrelvir and ritonavir	1 kit in 1 blister pack	2,544
00069034506	nirmatrelvir and ritonavir	1 kit in 1 blister pack	558
00069110104	nirmatrelvir and ritonavir	1 kit in 1 blister pack	318
00069208501	nirmatrelvir and ritonavir	4 tablet, film coated in 1 blister pack	N/A
00069134501	nirmatrelvir and ritonavir	2 tablet, film coated in 1 blister pack	N/A
00069208502	nirmatrelvir and ritonavir	2 tablet, film coated in 1 blister pack	N/A
00069308501	nirmatrelvir and ritonavir	2 tablet in 1 blister pack	N/A
00006505506	molnupiravir	40 capsule in 1 bottle, plastic	273,790
00006505509	molnupiravir	40 capsule in 1 bottle, plastic	N/A
00006505507	molnupiravir	40 capsule in 1 bottle, plastic	N/A

eMethods Table B. CPT/HCPCS codes used to identify IV COVID-19 therapies in the Carrier and institutional revenue files

CPT/HCPCS	Description	Claims (n), 2020-2022
M0239	Bamlanivimab-xxxx infusion[a]	132,380
M0245	Bamlan and etesev infusion	95,167
M0246	Bamlan and etesev infus home	1,791
Q0239	Bamlanivimab-xxxx[a]	21,926
Q0245	Bamlanivimab and etesevima	26,457
M0222	Bebtelovimab injection	148,512
M0223	Bebtelovimab injection home	9,646
Q0222	Bebtelovimab 175 mg	59,199
M0240	Casiri and imdev repeat	6,234
M0241	Casiri and imdev repeat hm	1,146
M0243	Casirivi and imdevi inj	456,590
M0244	Casirivi and imdevi inj hm	17,092
Q0240	Casirivi and imdevi 600mg	4,911
Q0243	Casirivimab and imdevimab	31,643
Q0244	Casirivi and imdevi 1200 mg	61,385
J0248	remdesivir	49,206
M0247	Sotrovimab infusion	75,798
M0248	Sotrovimab inf, home admin	3,224
Q0247	Sotrovimab	24,312

[a] We believe bamlanivimab-xxxx refers to "monotherapy" (versus bamlanivimab with etesevimab)

eMethods Table C. CPT/HCPCs codes used to identify molecular/antigen COVID-19 testing

CPT/HCPCS	Description	Claims (n), 2020-2022
Molecular Tests		
U0001	2019-ncov diagnostic p	56,536
U0002	covid-19 lab test non-cdc	948,730
U0003	cov-19 amp prb hgh thruput	10,729,948
U0004	cov-19 test non-cdc hgh thru	3,041,703
U0005	infec agen detec ampli probe	11,845,690
0202U	nfct ds 22 trgt sars-cov-2	157,031
0223U	nfct ds 22 trgt sars-cov-2	3,980
0225U	nfct ds dna&rna 21 sarscov2	7,106
0240U	nfct ds vir resp rna 3 trgt	86,990
0241U	nfct ds vir resp rna 4 trgt	822,717
87635	sars-cov-2 covid-19 amp prb	2,598,684
87636	sarscov2 & inf a&b amp prb	703,549
87637	sarscov2&inf a&b&rsv amp prb	313,849
D0606	molecular testing for a public health related pathogen (dental code)	N/A
Antigen Tests		
87426	sarscov coronavirus ag ia	2,955,963
87428	sarscov & inf vir a&b ag ia	611,330
87811	sars-cov-2 covid19 w/optic	1,029,341
D0604	antigen testing for any public health related pathogen (dental code)	N/A

eMethods Table D. CPT/HCPCS codes used to identify COVID-19 vaccinations

CPT/HCPCS	Description	Claims (n), 2020-2022
91302	AstraZeneca Covid-19 Vaccine	< 11
0021A	AstraZeneca Covid-19 Vaccine Administration – First Dose	< 11
0022A	AstraZeneca Covid-19 Vaccine Administration – Second Dose	43
M0201	COVID-19 vaccine home admin	271,317
91303	Janssen Covid-19 Vaccine (Aged 18 years and older)[3]	101,863
0031A	Janssen Covid-19 Vaccine Administration - First Dose[3]	1,483,205
0034A	Janssen Covid-19 Vaccine Administration - Booster[3]	143,901
91301	Moderna Covid-19 Vaccine (Aged 12 years and older) (Red Cap)	1,738,582
91306	Moderna Covid-19 Vaccine (Aged 18 years and older) (Red Cap) (Low Dose)	276,133
91309	Moderna Covid-19 Vaccine (Aged 6 years through 11 years or aged 18 years and older) (Blue Cap with purple border) 50MCG/0.5ML[5]	7,992
91311	Moderna Covid-19 Pediatric Vaccine (Aged 6 months through 5 years) (Blue Cap with magenta border) 250MCG/0.25ML	26

91313	Moderna COVID-19 Vaccine, Bivalent Product (Aged 12 years and older) (Dark Blue Cap with gray border)[6]	123,069
91314	Moderna COVID-19 Vaccine, Bivalent Product (Aged 6 years through 11 years) (Dark Blue Cap with gray border)	128
0011A	Moderna Covid-19 Vaccine (Red Cap) Administration – First Dose	12,271,347
0012A	Moderna Covid-19 Vaccine (Red Cap) Administration – Second Dose	11,575,825
0013A	Moderna Covid-19 Vaccine (Red Cap) Administration – Third Dose	3,428,726
0064A	Moderna Covid-19 Vaccine (Red Cap) (Low Dose) Administration - Booster	12,745,799
0091A	Moderna Covid-19 Pediatric Vaccine (Aged 6 years through 11 years) (Blue Cap with purple border) Administration - First dose	115
0092A	Moderna Covid-19 Pediatric Vaccine (Aged 6 years through 11 years) (Blue Cap with purple border) Administration - Second dose	12
0093A	Moderna Covid-19 Pediatric Vaccine (Aged 6 years through 11 years) (Blue Cap with purple border) Administration - Third dose	100
0094A	Moderna Covid-19 Vaccine (Aged 18 years and older) (Blue Cap with purple border) 50MCG/0.5ML Administration - Booster	46,977
0111A	Moderna Covid-19 Pediatric Vaccine (Aged 6 months through 5 years) (Blue Cap with magenta border) Administration - First dose	17
0112A	Moderna Covid-19 Pediatric Vaccine (Aged 6 months through 5 years) (Blue Cap with magenta border) Administration - Second dose	23
0113A	Moderna Covid-19 Pediatric Vaccine (Aged 6 months through 5 years) (Blue Cap with magenta border) Administration - Third dose	29
0134A	Moderna COVID-19 Vaccine, Bivalent (Aged 12 years and older) (Dark Blue Cap with gray border) Administration – Booster Dose[6]	3,485,308
0144A	Moderna COVID-19 Vaccine, Bivalent (Aged 6 years through 11 years) (Dark Blue Cap with gray border) Administration – Booster Dose	570
91304	Novavax Covid-19 Vaccine, Adjuvanted (Aged 12 years and older)	331
0041A	Novavax Covid-19 Vaccine, Adjuvanted Administration – First Dose	1,789
0042A	Novavax Covid-19 Vaccine, Adjuvanted Administration – Second Dose	983
0044A	Novavax Covid-19 Vaccine, Adjuvanted Administration - Booster	559
91300	Pfizer-BioNTech Covid-19 Vaccine (Aged 12 years and older) (Purple Cap)	1,893,174
91305	Pfizer-BioNTech Covid-19 Vaccine Pre-Diluted (Aged 12 years and older) (Gray Cap)	97,642
91307	Pfizer-BioNTech Covid-19 Pediatric Vaccine (Aged 5 years through 11 years) (Orange Cap)	202
91308	Pfizer-BioNTech Covid-19 Pediatric Vaccine (Aged 6 months through 4 years) (Maroon Cap)	12
91312	Pfizer-BioNTech COVID-19 Vaccine, Bivalent Product (Aged 12 years and older) (Gray Cap)	188,154
91315	Pfizer-BioNTech COVID-19 Vaccine, Bivalent Product (Aged 5 years through 11 years) (Orange Cap)	86
0001A	Pfizer-BioNTech Covid-19 Vaccine (Purple Cap) Administration – First Dose	12,634,252

0002A	Pfizer-BioNTech Covid-19 Vaccine (Purple Cap) Administration – Second Dose	11,997,243
0003A	Pfizer-BioNTech Covid-19 Vaccine (Purple Cap) Administration – Third Dose	4,839,851
0004A	Pfizer-BioNTech Covid-19 Vaccine (Purple Cap) Administration – Booster	8,312,146
0051A	Pfizer-BioNTech Covid-19 Vaccine Pre-Diluted (Gray Cap) Administration - First dose	1,072,652
0052A	Pfizer-BioNTech Covid-19 Vaccine Pre-Diluted (Gray Cap) Administration - Second dose	65,659
0053A	Pfizer-BioNTech Covid-19 Vaccine Pre-Diluted (Gray Cap) Administration - Third dose	171,309
0054A	Pfizer-BioNTech Covid-19 Vaccine Pre-Diluted (Gray Cap) Administration - Booster	2,347,252
0071A	Pfizer-BioNTech Covid-19 Pediatric Vaccine (Orange Cap) Administration - First dose	462
0072A	Pfizer-BioNTech Covid-19 Pediatric Vaccine (Orange Cap) Administration - Second dose	257
0073A	Pfizer-BioNTech Covid-19 Pediatric Vaccine (Orange Cap) Administration - Third dose	136
0074A	Pfizer-BioNTech Covid-19 Pediatric Vaccine (Orange Cap) Administration - Booster	265
0081A	Pfizer-BioNTech Covid-19 Pediatric Vaccine (Aged 6 months through 4 years) (Maroon Cap) Administration - First dose	33
0082A	Pfizer-BioNTech Covid-19 Pediatric Vaccine (Aged 6 months through 4 years) (Maroon Cap) Administration - Second dose	18
0083A	Pfizer-BioNTech Covid-19 Pediatric Vaccine (Aged 6 months through 4 years) (Maroon Cap) Administration - Third dose	23
0124A	Pfizer-BioNTech COVID-19 Vaccine, Bivalent (Gray Cap) Administration – Booster Dose	4,920,886
0154A	Pfizer-BioNTech COVID-19 Vaccine, Bivalent Product (Aged 5 years through 11 years) (Orange Cap) Administration – Booster Dose	535

© 2024 Wilcock AD et al. *JAMA Health Forum.*

eMethods 2. Model specification

Adjusted Odds-Ratios

Adjusted odds ratios shown in Figure 2 and reported in eTables 2 and 5 were estimated using logistic regression with clustered standard errors at the hospital referral region level (based on beneficiary residence zip).

Model specification was the following

$$Outcome_i = \beta_0 + X_i\tau + \varepsilon_i$$

- $Outcome_i$ is the outcome for beneficiary i
- ε_i is the error with hospital referral region level clustering
- X_i are beneficiary characteristics:
    - Age indicators (<40, 40-49, 50-59, 60-64, 65-69, 70-74, 75-79, 80-84, 85-89, and 90+) as of January 1 2022
    - Female indicator
    - Race/ethnicity indicators (Asian, black, Hispanic, non-white, and other (American Indian, Pacific Islander, multi race or unknown))
    - Metro residence indicator set equal to 1 if the bene's residence Zip code is located within Rural-Urban Commuting Areas (RUCAs) 1-3 (i.e., metropolitan area or "urban"), and 0 otherwise (i.e., non-metropolitan area or "rural")
    - Medicaid eligibility status (dual eligible or not) in any month of 2022
    - Original entitlement reason indicators (age 65+, disability or end-stage renal disease)
    - Nursing home resident indicator set equal to 1 for any short or long-term nursing home day in 2022, and 0 otherwise (i.e., community resident)
    - Count of 27 chronic condition indicators (No information, 0, 1-3, 4-5, 6-9, and 10+)
        - Conditions were counted if they were identified prior to the start of the 2022 and each was coded as 1 if beneficiary i had it and 0 otherwise
        - We included an indicator for patients without chronic condition information
        - For those with information, conditions included: Alzheimer's disease, Alzheimer's disease and related disorders or senile dementia, anemia, asthma, atrial fibrillation, benign prostatic hyperplasia, breast cancer, cataract, chronic kidney disease, chronic obstructive pulmonary disease, colorectal cancer, depression, diabetes, endometrial cancer, glaucoma, heart failure, hip or pelvic fracture, hyperlipidemia, hypertension, hypothyroidism, ischemic heart disease, lung cancer, osteoporosis, prostate cancer, acute myocardial infarction, rheumatoid arthritis, and stroke or transient ischemic attack.

© 2024 Wilcock AD et al. *JAMA Health Forum.*

    - Vaccination status indicator set equal to 1 for beneficiaries with at least 1 COVID-19 vaccine claim (see Methods Table D for codes) over the period 2020-2022, and 0 otherwise.
    - Indicators for contraindication level for Nirmatrelvir (i.e., Paxlovid),
        - Level 1, no contraindication ("none") or those with no history of liver/kidney disease, and no drug-drug interactions (Methods Table E)
        - Level 2, moderate contraindication or those with any tier 1 interactions (drugs listed in the monitor, adjust, or temporarily suspend concurrent medication sections of Methods Table E) and/or liver/kidney disease
        - Level 3, severe contraindication or those with any tier 2 drug interactions (drugs listed in the prescribe alternative COVID-19 therapy section of Methods Table E)

Geographic or Attributed Primary Care Practice Adjusted Models
We examined the effect of adding fixed effects for hospital referral region (HRR) or primary care practice (indexed by j) to a baseline linear regression model of any therapy use on beneficiary i characteristics. Baseline unadjusted effects, those with control variables added but without the addition of fixed effects, and each fixed effect model (HRR or practice) are provided in eTable 9 below. We also provide the % change of each coefficient from the fixed effect models compared to the adjusted model coefficients without fixed effects.

Model specification was the following

$$AnyTherapyUse_{ij} = \beta_0 + \delta_j + X_i\tau + \varepsilon_{ij}$$

- $AnyTherapyUse_{ij}$ is an indicator equal to 1 if beneficiary i used an oral or IV therapy in 2022
- ε_{ij} is the error with hospital referral region level clustering
- X_i are beneficiary characteristics (described above)
- δ_j is a fixed effect for beneficiary hospital referral region or attributed primary care practice (see eMethods 3 below for details on attribution)

Severity Risk Model
We defined a prognostic score for mortality within 21 days of a COVID-19 diagnosis ("COVID-19 severity risk") by counting up the incremental 21 day mortality probability associated with each patient characteristic. Expectations were the product of having a given characteristic and that characteristics' coefficient from a linear regression model of 21 day mortality fitted on a sample of patients diagnosed with COVID-19 in 2021. We limited our sample to beneficiaries with at least 180 days of continuous parts A/B enrollment prior to diagnosis through up to 21 days after. Beneficiaries enrolled in a Medicare Advantage plan during this period were not included. We also required beneficiaries to be continuously enrolled in part D Medicare in the 21 days before and after diagnosis to make them more comparable to our 2022 study sample (which we required to have 12 months of Part D coverage in 2022).

To better capture new incidence of COVID-19 (as opposed to documenting or treating prior infections) we limited our sample to COVID-19 cases that did not have any preceding COVID-19 diagnoses in the prior 180 days, and among those only kept cases with a primary

© 2024 Wilcock AD et al. *JAMA Health Forum.*

diagnosis for COVID-19. After making these restrictions, our sample included 1,170,947 COVID-19 cases from January 1 2021 through December 31 2021.

Using this sample, we employed the following model specification to generate the coefficients we used to calculate beneficiary risk scores in 2022.

Model specification was the following:

$$21dayMortality_i = \beta_0 + X_i\tau + \varepsilon_i$$

- $21dayMortality_i$ is a dichotomous indicator for whether or not patient i died within 21 days of their COVID-19 diagnosis from any cause (i.e., all-cause 21 day mortality)
- β_0 is a constant
- ε_i is random error
- X_i are beneficiary demographics, including:
  - Age indicators (<40, 40-49, 50-59, 60-64, 65-69, 70-74, 75-79, 80-84, 85-89, and 90+) as of diagnosis date
  - Female indicator
  - Race/ethnicity indicators (Asian, black, Hispanic, non-white, and other (American Indian, Pacific Islander, multi race or unknown))
  - Metro residence indicator set equal to 1 if the bene's residence Zip code is located within Rural-Urban Commuting Areas (RUCAs) 1-3 (i.e., metropolitan area or "urban"), and 0 otherwise (i.e., non-metropolitan area or "rural")
  - Medicaid eligibility status (dual eligible or not) in any month 1 year prior to diagnosis
  - Original entitlement reason indicators (age 65+, disability or end-stage renal disease)
  - 27 chronic condition indicators
    - Conditions were included if they were identified prior to the start of the 2021 and each was coded as 1 if beneficiary i had it and 0 otherwise
    - We included an indicator for patients without chronic condition information
    - For those with information, conditions included: Alzheimer's disease, Alzheimer's disease and related disorders or senile dementia, anemia, asthma, atrial fibrillation, benign prostatic hyperplasia, breast cancer, cataract, chronic kidney disease, chronic obstructive pulmonary disease, colorectal cancer, depression, diabetes, endometrial cancer, glaucoma, heart failure, hip or pelvic fracture, hyperlipidemia, hypertension, hypothyroidism, ischemic heart disease, lung cancer, osteoporosis, prostate cancer, acute myocardial infarction, rheumatoid arthritis, and stroke or transient ischemic attack.
  - Vaccination status indicator set equal to 1 for beneficiaries with at least 1 COVID-19 vaccine claim (see Methods Table D for codes) prior to their COVID-19 diagnosis date, and 0 otherwise.
  - 37 other CCW Condition Indicators
    - Conditions were included if they were identified prior to the start of the 2021 and each was coded as 1 if beneficiary i had it and 0 otherwise

© 2024 Wilcock AD et al. *JAMA Health Forum.*

- Conditions included: ADHD/ Conduct Disorders/ Hyperkinetic Syndrome, Alcohol Use Disorders, Anxiety Disorders, Autism Spectrum Disorders, Bipolar Disorder, Traumatic Brain Injury/ Brain Damage Disorders, Cerebral Palsy, Cystic Fibrosis/Metabolic Developmental Disorders, Depressive Disorders, Drug Use Disorders, Epilepsy, Fibromyalgia/Chronic Pain/Fatigue, Deafness /Hearing Impairment, Viral Hepatitis, HIV/AIDS, Intellectual Disabilities, Learning Disabilities, Leukemias/Lymphomas, Liver Disease/Cirrhosis/Other Liver Conditions, Migraine/Chronic Headache, Mobility Impairments, Multiple Sclerosis/Transverse Myelitis, Muscular Dystrophy, Obesity, Other Developmental Delays, Opioid Use Disorder, Personality Disorders, Post-Traumatic Stress Disorder, Peripheral Vascular Disease, Sickle Cell Disease, Schizophrenia, Schizophrenia/Other Psychotic Disorders, Spina Bifida/Other Congenital Nervous System Anomalies, Spinal Cord Injury, Tobacco Use, Ulcers, Blindness/Visual Impairment.

eTable 1 contains the coefficients we estimated to predict severity risk. The C-statistic for our model (obtained via the area under the ROC, non-parametrically) was 0.75.

© 2024 Wilcock AD et al. *JAMA Health Forum.*

eMethods 3. Primary care practice attribution and nirmatrelvir contraindications

Primary Care Attribution
We identified 2020-2021 primary care visits in the part B Carrier claim files as E&M visits (using the BETOS E&M grouper of CPT/HCPCS codes) delivered by a physician with a provider specialty code for general practice, internal medicine, family medicine, osteopathic therapist, pediatric medicine, geriatric medicine, or advanced nursing practice.

We considered tax identification numbers (TINs) to be "practices", and we attributed beneficiaries to the practice that accounted for the plurality of primary care E&M visits in a prior year. First, we assigned beneficiaries to the plurality practice based on their primary care E&M visits in 2021. If the beneficiary did not have at least 1 primary care visit from 2021 from which to make the assignment, we then assigned based on their visits in 2020. For beneficiaries in our 2022 sample that remained unassigned (13.7% of total), we grouped them under their 5-digit residential zip code as their assigned practice.

Contraindication Levels for Nirmatrelvir
As described in the Methods, nirmatrelvir has multiple potential contraindications, including specific clinical conditions (kidney or liver disease) or drug-drug interactions, and these contraindications could be a major impediment to its use.[25] Using treatment guidelines,[26] we identified two tiers of drug safety risk for nirmatrelvir: tier 1 drugs, which need to be closely monitored or those that require dosage adjustment; and tier 2 drugs, which are contraindicated and must be held if nirmatrelvir is prescribed.

For each beneficiary we identified

- Prior history of kidney or liver disease using their CCW chronic and other conditions indicators for chronic kidney disease and Liver Disease/Cirrhosis/Other Liver Conditions
- And, whether they filled prescriptions with ingredient believed to cause an adverse interaction with nirmatrelvir
    - For which, we searched their part D event histories from October 2021 through December 2022 to see if they ever had NDC codes linked to one of the ingredients listed in Methods Table E below

<table>
<tr><th>Contraindication Level</th><th>Interaction Tier</th><th>Description</th><th>Liver/Kidney Disease</th></tr>
<tr><td>1: None</td><td>No interactions</td><td>--</td><td>No prior history</td></tr>
<tr><td rowspan="3">2: Moderate</td><td rowspan="3">Tier 1</td><td>Monitor for adverse effects</td><td rowspan="3">Has prior history</td></tr>
<tr><td>Adjust dose and monitor</td></tr>
<tr><td>Temporarily withhold</td></tr>
<tr><td>3: Severe</td><td>Tier 2</td><td>Prescribe alternative COVID-19 therapy</td><td>No prior history or Has prior history</td></tr>
</table>

Then, we assigned each beneficiary to one of three contraindication levels described in the grid above based on their history of prescription fills and kidney/liver disease. For the interaction

tiers, we obtained from treatment guidelines[26] the names of generic ingredients in each tier and identified the NDC codes that fell under each ingredient using the part D drug characteristic file. eMethods Table E describes the number of NDCs we were able to find under each generic ingredient name. Some ingredients such as dihydroergocornine did not correspond to any NDCs but we include them in the table to accurately describe our search.

© 2024 Wilcock AD et al. *JAMA Health Forum.*

eMethods Table E. Ingredients and unique National Drug Codes (NDCs) in the Part D Drug Characteristics File

**Prescribe Alternative COVID-19 Therapy**		
Medication Name	Generic Ingredient Name (GNN) in Part D Drug Characteristics File	# NDCs
AMIODARONE	AMIODARONE HCL	48
BOSENTAN	BOSENTAN	18
BROMOCRIPTINE	BROMOCRIPTINE MESYLATE	17
CARBAMAZEPINE	CARBAMAZEPINE	72
CLOPIDOGREL	CLOPIDOGREL BISULFATE	73
CLOZAPINE	CLOZAPINE	97
DIHYDROERGOCORNINE		0
DIHYDROERGOCRISTINE		0
DIHYDROERGOCRYPTINE		0
DIHYDROERGOTAMINE		
ERGOTAMINE	DIHYDROERGOTAMINE MESYLATE	12
DISOPYRAMIDE	DISOPYRAMIDE PHOSPHATE	13
DOFETILIDE	DOFETILIDE	37
DRONEDARONE	DRONEDARONE HCL	2
EPLERENONE	EPLERENONE	30
ERGONOVINE	METHYLERGONOVINE MALEATE	10
ERGOTAMINE	ERGOTAMINE TARTRATE/CAFFEINE	5
FLECAINIDE	FLECAINIDE ACETATE	34
GLECAPREVIR PIBRENTASVIR	GLECAPREVIR/PIBRENTASVIR	2
IMACAFTOR		0
IVABRADINE	IVABRADINE HCL	3
IVACAFTOR LUMACAFTOR	LUMACAFTOR/IVACAFTOR	1
LURASIDONE	LURASIDONE HCL	11
METHYSERGIDE		0
MIDAZOLAM	MIDAZOLAM HCL	47
PERGOLIDE		0
PHENOBARBITAL	PHENOBARBITAL	107
PHENYTOIN	FOSPHENYTOIN SODIUM	69
PIMOZIDE	PIMOZIDE	2
PRIMIDONE	PRIMIDONE	31
PROPAFENONE	PROPAFENONE HCL	54
QUINIDINE	DEXTROMETHORPHAN HBR/QUINIDINE	5
RIFAMPIN	RIFAMPIN	30
RIFAPENTINE	RIFAPENTINE	3
SILDENAFIL	SILDENAFIL CITRATE	85
TADALAFIL	TADALAFIL	114
VARDENAFIL	VARDENAFIL HCL	13
VOCLOSPORIN		0
**Temporarily Withhold Concomitant Medication, if Clinically Appropriate**		
Medication Name	GNN in Part D Drug Characteristics File	# NDCs
ALFUZOSIN	ALFUZOSIN HCL	19
ALISKIREN	ALISKIREN/HYDROCHLOROTHIAZIDE	10
AMLODIPINE ATORVASTATIN	AMLODIPINE/ATORVASTATIN	55
ATORVASTATIN	ATORVASTATIN CALCIUM	245
AVANAFIL	AVANAFIL	1
COLCHICINE	COLCHICINE	36
ELETRIPTAN	ELETRIPTAN HYDROBROMIDE	21
ERYTHROMYCIN	ERYTHROMYCIN BASE	86

© 2024 Wilcock AD et al. *JAMA Health Forum.*

EVEROLIMUS	EVEROLIMUS	33
FINERENONE		0
FLIBANSERIN		0
LOMITAPIDE	LOMITAPIDE MESYLATE	5
LOVASTATIN	LOVASTATIN	64
NALOXEGOL	NALOXEGOL OXALATE	6
RANOLAZINE	RANOLAZINE	27
RIMEGEPANT	RIMEGEPANT SULFATE	1
RIVAROXABAN	RIVAROXABAN	17
ROSUVASTATIN	ROSUVASTATIN CALCIUM	125
SALMETEROL	SALMETEROL XINAFOATE	29
SILODOSIN	SILODOSIN	30
SIMVASTATIN	SIMVASTATIN	280
SIROLIMUS	SIROLIMUS	23
SUVOREXANT	SUVOREXANT	8
TACROLIMUS	TACROLIMUS	86
TICAGRELOR	TICAGRELOR	3
TRIAZOLAM	TRIAZOLAM	10
UBROGEPANT	UBROGEPANT	4
VORAPAXAR	VORAPAXAR SULFATE	2
**Adjust Concomitant Medication Dose and Monitor for Adverse Effects**		
Medication Name	GNN in Part D Drug Characteristics File	# NDCs
ALMOTRIPTAN	ALMOTRIPTAN MALATE	6
ALPRAZOLAM	ALPRAZOLAM	111
APIXABAN	APIXABAN	6
ARIPIPRAZOLE	ARIPIPRAZOLE	219
BREXIPIPRAZOLE		0
BUSPIRONE	BUSPIRONE HCL	138
CARIPRAZINE	CARIPRAZINE HCL	9
CHLORDIAZEPOXIDE	CHLORDIAZEPOXIDE HCL	23
CILOSTAZOL	CILOSTAZOL	17
CLARITHROMYCIN	CLARITHROMYCIN	22
CLOBAZAM	CLOBAZAM	45
CLONAZEPAM	CLONAZEPAM	106
CLORAZEPATE	CLORAZEPATE DIPOTASSIUM	11
CYCLOSPORINE	CYCLOSPORINE, MODIFIED	39
DABIGATRAN	DABIGATRAN ETEXILATE MESYLATE	10
DARIFENACIN	DARIFENACIN HYDROBROMIDE	29
DEXAMETHASONE	DEXAMETHASONE	98
DIAZEPAM	DIAZEPAM	66
DIGOXIN	DIGOXIN	62
EDOXABAN	EDOXABAN TOSYLATE	7
ELEXACAFTOR TEZACAFTOR	ELEXACAFTOR/TEZACAFTOR/IVACAFT	1
ELUXADOLINE	ELUXADOLINE	2
ESTAZOLAM	ESTAZOLAM	4
FEDRATINIB	FEDRATINIB DIHYDROCHLORIDE	1
FENTANYL	FENTANYL	120
FLURAZEPAM	FLURAZEPAM HCL	2
HYDROCODONE	HYDROCODONE/ACETAMINOPHEN	256
ILOPERIDONE	ILOPERIDONE	8
ITRACONAZOLE	ITRACONAZOLE	22
IVACAFTOR	IVACAFTOR	2
IVACAFTOR TEZACAFTOR	TEZACAFTOR/IVACAFTOR	1
KETOCONAZOLE	KETOCONAZOLE	44
LUMATEPERONE	LUMATEPERONE TOSYLATE	1

© 2024 Wilcock AD et al. *JAMA Health Forum.*

MARAVIROC	MARAVIROC	4
MEXILETINE	MEXILETINE HCL	12
OXYCODONE	OXYCODONE HCL/ACETAMINOPHEN	307
PIMAVANSERIN	PIMAVANSERIN TARTRATE	3
QUETIAPINE	QUETIAPINE FUMARATE	259
RIFABUTIN	OMEPRAZOLE/AMOXICILL/RIFABUTIN	6
RIOCIGUAT	RIOCIGUAT	5
RUXOLITINIB	RUXOLITINIB PHOSPHATE	5
SAXAGLIPTIN	SAXAGLIPTIN HCL/METFORMIN HCL	9
SOLIFENACIN	SOLIFENACIN SUCCINATE	74
TAMSULOSIN	DUTASTERIDE/TAMSULOSIN HCL	51
TOFACITINIB	TOFACITINIB CITRATE	5
TRAZODONE	TRAZODONE HCL	89
UPADACITINIB	UPADACITINIB	1
**Continue Concomitant Medication and Monitor for Adverse Effects**		
Medication Name	GNN in Part D Drug Characteristics File	# NDCs
AMLODIPINE	AMLODIPINE BESYLATE	342
AMLODIPINE VALSARTAN	AMLODIPINE/VALSARTAN/HCTHIAZID	73
BRINCIDOFOVIR		0
BUPRENORPHINE	BUPRENORPHINE	132
COBICISTAT	DARUNAVIR/COBICISTAT	3
DILTIAZEM	DILTIAZEM HCL	259
DOXAZOSIN	DOXAZOSIN MESYLATE	80
FELODIPINE	FELODIPINE	49
GLYBURIDE	GLYBURIDE	78
HALOPERIDOL	HALOPERIDOL	125
HYDROMORPHONE	HYDROMORPHONE HCL	91
HYDROXYZINE	HYDROXYZINE PAMOATE	117
ISAVUCONAZOLE		0
METHADONE	METHADONE HCL	40
MIRTAZAPINE	MIRTAZAPINE	103
MORPHINE	MORPHINE SULFATE	187
NIFEDIPINE	NIFEDIPINE	79
OXYBUTYNIN	OXYBUTYNIN CHLORIDE	114
POSACONAZOLE	POSACONAZOLE	6
RISPERIDONE	RISPERIDONE MICROSPHERES	174
SACUBITRIL VALSARTAN	SACUBITRIL/VALSARTAN	11
TERAZOSIN	TERAZOSIN HCL	39
TRAMADOL	TRAMADOL HCL	86
VALSARTAN	VALSARTAN/HYDROCHLOROTHIAZIDE	139
VERAPAMIL	TRANDOLAPRIL/VERAPAMIL HCL	92
VORICONAZOLE	VORICONAZOLE	40
WARFARIN	WARFARIN SODIUM	205
ZIPRASIDONE	ZIPRASIDONE HCL	70
ZOLMITRIPTAN	ZOLMITRIPTAN	35
ZOLPIDEM	ZOLPIDEM TARTRATE	65

Drug interactions defined using NIH treatment guidelines available at https://www.covid19treatmentguidelines.nih.gov/therapies/antivirals-including-antibody-products/ritonavir-boosted-nirmatrelvir--paxlovid-/paxlovid-drug-drug-interactions/

© 2024 Wilcock AD et al. *JAMA Health Forum.*

eMethods 4. Details on simulated reallocation of nirmatrelvir

Specifying adverse events as a function of COVID-19 cases and treatments

For any risk group *k*, we can express the number of adverse COVID-related events *E* (*e.g.*, hospitalizations or deaths) in terms of the number of COVID-19 cases *C* and the number of treatments *T* :

$$E_k \;=\; (C_k - T_k)\, P(E_k|\, C_k) \;+\; T_k(1-\sigma)\, P(E_k|\, C_k) \qquad (1)$$

Here, P(*E*|*C*) is the probability that the event *E* occurs given a symptomatic COVID-19 case *C*, and σ is the treatment effectiveness (*i.e.*, the proportion reduction in risk due to treatment). This equation states that the number of adverse events *E* is equal to (a) the number of untreated COVID-19 cases times the baseline probability that the event occurs given a case, plus (b) the number of treated COVID cases times the treatment-adjusted probability that the event occurs given a case.

Accounting for imperfect ascertainment

The total number of COVID-19 cases, C_k, is not directly observed. Because of this, it is necessary to adjust Equation (1) for imperfect case ascertainment. Let c_k (lowercase) denote the observed number of cases and a_k denote the ascertainment rate, so that

$$a_k\, C_k \;=\; c_k \qquad (2)$$

Thus, we can re-write the Equation (1) as

$$E_k \;=\; (c_k/a_k - T_k)\, P(E_k|\, c_k, a_k) \;+\; T_k(1-\sigma)\, P(E_k|\, c_k, a_k) \qquad (3)$$

We can further simplify Equation (3):

$$E_k \;=\; P(E_k|\, c_k, a_k)\, [c_k/a_k \;-\; \sigma\, T_k] \qquad (4)$$

Equation (4) now specifies the number of adverse outcomes *E* as a function of the observed cases *c*, the ascertainment rate *a*, the number of treatments *T*, and the treatment efficacy σ.

Accounting for unknowns

If we assume that we observe all treatments *T* and all adverse events *E*, we are left with two key unknowns:

- a_k, the case ascertainment rate. This may vary across risk groups *k*.
- σ, the treatment efficacy. We assume that this is constant across risk groups, though this assumption can be relaxed.

By making informed guesses about these values, it is possible to calculate P(E_k| c_k, a_k) (the risk of adverse outcomes) from the available data. For our analysis, we consider a range of ascertainment rates from 50% to 75%. We set the treatment efficacy based on literature values.

Assessing different treatment distributions across risk groups

© 2024 Wilcock AD et al. *JAMA Health Forum.*

With a slight adjustment to Equation (1), it is possible to estimate the number of adverse events that would have occurred with a different number of treatments:

$$\widetilde{E}_k = P(E_k | c_k, a_k) [c_k/a_k - \sigma \widetilde{T}_k] \quad (5)$$

Here, $\widetilde{T}_k$ is the counterfactual (adjusted) number of treatments in group k and $\widetilde{E}_k$ is the expected number of adverse events that would have occurred with $\widetilde{T}_k$ treatments.

We re-allocated treatments across groups in proportion to the estimated baseline (non-treatment) risk of adverse outcomes, so that the total number of treatments given is the same as the actual number of treatments given, but each risk group instead receives a share of those treatments in proportion to their baseline risk of adverse outcomes (hospitalization or death). In this scenario, the total number of treatments in the population remains constant. Because of this, outcomes may worsen for some risk groups if treatments are re-allocated from that group, due to lower baseline risk, and given instead to other, higher-risk groups.

For the present analysis, we considered treatment with nirmatrelvir. We assumed a $\sigma = 40\%$ reduction in the risk of hospitalization and a $\sigma = 70\%$ reduction in mortality risk. Using these values, we calculated the counterfactual change in adverse outcomes (hospitalizations or mortality) under the treatment redistribution scenarios outlined above, under a range of case ascertainment rates a from 50% to 75%.

© 2024 Wilcock AD et al. *JAMA Health Forum.*

eTable 1. Mortality following COVID-19 Diagnosis Risk Model Coefficients

	Coef.	SE	P value	95% Confidence Interval Lower	Upper
**Patient Characteristics**					
Age Less than 40	-0.0470	0.00152	0.0000	-0.0500	-0.0440
Age 40 to 49	-0.0370	0.00143	0.0000	-0.0398	-0.0342
Age 50 to 59	-0.0250	0.00116	0.0000	-0.0273	-0.0227
Age 60 to 64	-0.0111	0.00126	0.0000	-0.0136	-0.0087
Age 65 to 69	ref				
Age 70 to 74	0.0109	0.000691	0.0000	0.0095	0.0122
Age 75 to 79	0.0267	0.000803	0.0000	0.0251	0.0282
Age 80 to 84	0.0476	0.000933	0.0000	0.0458	0.0494
Age 85 to 89	0.0811	0.0011	0.0000	0.0790	0.0833
Age 90 plus	0.1280	0.00124	0.0000	0.1260	0.1310
Female	-0.0196	0.000559	0.0000	-0.0207	-0.0185
Male	ref				
Asian	0.0108	0.0016	0.0000	0.0077	0.0139
Black	0.0021	0.000831	0.0104	0.0005	0.0038
Hispanic	0.0078	0.000896	0.0000	0.0061	0.0096
White	ref				
Other Race	0.0003	0.00121	0.8320	-0.0021	0.0026
Urban Residence	ref				
Rural Residence	0.0055	0.000484	0.0000	0.0046	0.0064
Non-Dual	ref				
Dual	0.0117	0.00059	0.0000	0.0106	0.0129
Entitlement Age	ref				
Entitlement Disability	0.0165	0.000748	0.0000	0.0150	0.0179
Entitlement ESRD	0.0448	0.00193	0.0000	0.0410	0.0485
No Vaccination	ref				
Vaccination	-0.0333	0.000457	0.0000	-0.0342	-0.0324
**CCW Chronic Condition Indicators**					
CCW No Info	0.0009	0.00108	0.3850	-0.0012	0.0031
Any Dementia	0.0185	0.000811	0.0000	0.0169	0.0201
Alzheimer's	0.0168	0.00116	0.0000	0.0145	0.0191
Acute Myocardial Infarction	0.0169	0.00105	0.0000	0.0148	0.0190
Anemia	0.0040	0.000533	0.0000	0.0029	0.0050
Asthma	-0.0076	0.000623	0.0000	-0.0088	-0.0064
Atrial Fibrillation	0.0042	0.000675	0.0000	0.0028	0.0055
Cataract	-0.0043	0.000534	0.0000	-0.0054	-0.0033
Cogestive Heart Failure	0.0180	0.000614	0.0000	0.0167	0.0192
Chronic Kidney Disease	0.0189	0.000542	0.0000	0.0178	0.0199
Endocrine Cancer	-0.0018	0.00221	0.4270	-0.0061	0.0026

© 2024 Wilcock AD et al. *JAMA Health Forum.*

Breast Cancer	-0.0041	0.00101	0.0000	-0.0061	-0.0022
Colon Cancer	0.0021	0.00144	0.1450	-0.0007	0.0049
Lung Cancer	0.0263	0.00192	0.0000	0.0225	0.0300
Prostate Cancer	-0.0041	0.00107	0.0002	-0.0062	-0.0020
Chronic Obstructive Pulmonary Disease	0.0126	0.00058	0.0000	0.0114	0.0137
Depression	0.0025	0.000817	0.0026	0.0009	0.0041
Diabetes	0.0058	0.000529	0.0000	0.0047	0.0068
Glaucoma	-0.0048	0.000562	0.0000	-0.0060	-0.0037
Hip Fracture	0.0120	0.00128	0.0000	0.0095	0.0145
Hyperlipidemia	-0.0080	0.000619	0.0000	-0.0092	-0.0068
Benign Prostatic Hyperplasia	-0.0021	0.000701	0.0030	-0.0035	-0.0007
Hypertension	0.0004	0.000639	0.4870	-0.0008	0.0017
Thyroid Disease	-0.0010	0.000513	0.0598	-0.0020	0.0000
Ischemic Heart Disease	-0.0026	0.000549	0.0000	-0.0037	-0.0015
Osteoporosis	-0.0075	0.00063	0.0000	-0.0087	-0.0062
Arthritis	-0.0050	0.000538	0.0000	-0.0061	-0.0040
Stroke	0.0016	0.000721	0.0227	0.0002	0.0031
**Other CCW Condition Indicators**		0			
ADHD/ Conduct Disorders/ Hyperkinetic Syndrome	-0.0040	0.00141	0.0043	-0.0068	-0.0013
Alcohol Use Disorders	-0.0037	0.00111	0.0009	-0.0059	-0.0015
Anxiety Disorders	-0.0024	0.000576	0.0000	-0.0036	-0.0013
Autism Spectrum Disorders	-0.0013	0.00289	0.6600	-0.0069	0.0044
Bipolar Disorder	-0.0039	0.000987	0.0001	-0.0059	-0.0020
Traumatic Brain Injury/ Brain Damage Disorders	-0.0041	0.00177	0.0215	-0.0075	-0.0006
Cerebral Palsy	-0.0062	0.00249	0.0127	-0.0111	-0.0013
Cystic Fibrosis/Metabolic Developmental Disorders	-0.0044	0.00134	0.0010	-0.0071	-0.0018
Depressive Disorders	-0.0015	0.000861	0.0736	-0.0032	0.0001
Drug Use Disorders	-0.0019	0.0011	0.0916	-0.0040	0.0003
Epilepsy	0.0013	0.00105	0.2100	-0.0007	0.0034
Fibromyalgia/Chronic Pain/Fatigue	-0.0059	0.000513	0.0000	-0.0069	-0.0049
Deafness /Hearing Impairment	-0.0052	0.000644	0.0000	-0.0064	-0.0039
Viral Hepatitis	-0.0021	0.00144	0.1420	-0.0050	0.0007
HIV/AIDS	-0.0139	0.00306	0.0000	-0.0198	-0.0079
Intellectual Disabilities	0.0052	0.00158	0.0011	0.0021	0.0083
Learning Disabilities	0.0020	0.00271	0.4540	-0.0033	0.0074
Leukemias/Lymphomas	0.0262	0.00137	0.0000	0.0236	0.0289
Liver Disease/Cirrhosis/Other Liver Conditions	-0.0010	0.000684	0.1460	-0.0023	0.0003
Migraine/Chronic Headache	-0.0065	0.000801	0.0000	-0.0081	-0.0050
Mobility Impairments	0.0071	0.00104	0.0000	0.0051	0.0092
Multiple Sclerosis/Transverse Myelitis	0.0012	0.00213	0.5690	-0.0030	0.0054

© 2024 Wilcock AD et al. *JAMA Health Forum.*

Muscular Dystrophy	0.0216	0.00595	0.0003	0.0099	0.0332
Obesity	0.0076	0.000502	0.0000	0.0066	0.0086
Other Developmental Delays	-0.0029	0.00293	0.3270	-0.0086	0.0029
Opioid Use Disorder	0.0044	0.00123	0.0003	0.0020	0.0068
Personality Disorders	-0.0049	0.00121	0.0000	-0.0073	-0.0026
Post-Traumatic Stress Disorder	-0.0012	0.00145	0.4020	-0.0041	0.0016
Peripheral Vascular Disease	0.0035	0.000603	0.0000	0.0023	0.0047
Sickle Cell Disease	0.0012	0.00685	0.8610	-0.0122	0.0146
Schizophrenia	-0.0158	0.00172	0.0000	-0.0192	-0.0125
Schizophrenia/Other Psychotic Disorders	0.0032	0.00131	0.0147	0.0006	0.0058
Spina Bifida/Other Congenital Nervous System Anomalies	-0.0040	0.00306	0.1910	-0.0100	0.0020
Spinal Cord Injury	0.0048	0.00174	0.0054	0.0014	0.0083
Tobacco Use	0.0100	0.000685	0.0000	0.0087	0.0114
Ulcers	0.0189	0.000807	0.0000	0.0173	0.0205
Blindness/Visual Impairment	0.0121	0.00178	0.0000	0.0086	0.0156
Constant	0.0367	0.0007	0.0000	0.0353	0.0382
Observations	1,170,947				
R^2	0.051				

See eMethods 2 for description of the COVID-19 severity risk model above.

© 2024 Wilcock AD et al. *JAMA Health Forum.*

eTable 2. Rates of COVID-19 therapy by demographics among patients with a COVID-19 diagnosis in 2022

		Beneficiaries with COVID-19	Any COVID-19 Therapy		Oral COVID-19 Therapy		IV COVID-19 Therapy	
		N (col %)	Row %	Adj. OR (95% CI)	Row %	Adj. OR (95% CI)	Row %	Adj. OR (95% CI)
	Total, No.	3,715,664	23.0		18.1		5.2	
Age	Under 40	1.7	12.5	0.73 (0.70, 0.76)	8.5	0.66 (0.63, 0.68)	4.1	1.08 (0.99, 1.18)
	40-49	1.9	15.1	0.87 (0.85, 0.90)	10.5	0.83 (0.81, 0.86)	4.8	1.07 (0.99, 1.15)
	50-59	3.5	15.9	0.91 (0.89, 0.94)	11.4	0.91 (0.89, 0.94)	4.7	0.96 (0.92, 1.01)
	60-64	3.1	17.1	0.93 (0.91, 0.95)	12.5	0.93 (0.91, 0.95)	4.8	0.96 (0.92, 1.01)
	65-69	24.0	25.0	Ref	20.7	Ref	4.6	Ref
	70-74	24.3	25.5	1.00 (0.99, 1.01)	20.6	1.01 (1.00, 1.02)	5.2	0.97 (0.95, 0.99)
	75-79	17.9	24.4	0.97 (0.96, 0.99)	19.0	0.98 (0.96, 1.00)	5.7	0.98 (0.95, 1.00)
	80-84	11.7	22.7	0.98 (0.96, 1.00)	16.9	0.97 (0.94, 0.99)	6.1	1.03 (1.00, 1.06)
	85-89	6.9	20.3	0.98 (0.95, 1.00)	14.6	0.95 (0.92, 0.98)	6.0	1.07 (1.04, 1.11)
	90+	5.0	16.7	0.90 (0.88, 0.93)	11.6	0.87 (0.84, 0.90)	5.3	1.05 (1.00, 1.1)
Sex	Female	58.4	22.7	0.98 (0.97, 0.99)	18.0	1 (0.99, 1.01)	4.9	0.93 (0.92, 0.94)
	Male	41.6	23.5	Ref	18.2	Ref	5.6	Ref
Race	Asian	3.2	22.1	0.96 (0.88, 1.05)	19.4	1.10 (1.00, 1.21)	2.9	0.58 (0.50, 0.67)
	Black	5.9	13.7	0.68 (0.66, 0.71)	10.6	0.74 (0.71, 0.76)	3.2	0.60 (0.56, 0.65)
	Hispanic	5.2	17.6	0.86 (0.81, 0.91)	13.9	0.93 (0.87, 0.99)	3.9	0.73 (0.62, 0.86)
	White	81.9	24.0	Ref	18.8	Ref	5.6	Ref
	Other Race[a]	3.8	24.6	1.01 (0.98, 1.03)	19.9	1.02 (0.99, 1.05)	5.0	0.97 (0.92, 1.01)
Geography	Urban	79.5	23.0	Ref	18.4	Ref	4.9	Ref
	Rural	20.5	23.1	1.07 (1.03, 1.12)	16.8	0.98 (0.95, 1.02)	6.5	1.31 (1.16, 1.49)
Medicaid Eligibility	Non-Duals	77.2	26.0	Ref	20.6	Ref	5.6	Ref
	Duals	22.8	13.1	0.65 (0.63, 0.68)	9.4	0.66 (0.64, 0.68)	3.8	0.72 (0.65, 0.80)
Original Reason for Medicare Eligibility	Age	80.7	24.5	Ref	19.6	Ref	5.2	Ref
	Disability	18.4	16.9	0.92 (0.91, 0.93)	12.2	0.89 (0.87, 0.90)	4.8	1.05 (1.01, 1.09)
	ESRD	0.9	16.8	0.96 (0.90, 1.03)	5.0	0.34 (0.32, 0.37)	12.2	3.05 (2.84, 3.27)
Institution	Community	86.4	25.3	Ref	20.0	Ref	5.6	Ref
	Nursing Home	13.6	8.6	0.35 (0.34, 0.36)	5.7	0.34 (0.32, 0.35)	3.0	0.47 (0.45, 0.50)
Chronic Condition Count[b]	No information	4.3	23.7	1.07 (1.05, 1.09)	19.8	1.05 (1.03, 1.07)	4.2	1.14 (1.09, 1.19)
	0	9.8	23.4	Ref	19.9	Ref	3.8	Ref
	1-3	15.3	24.5	1.04 (1.03, 1.06)	21.0	1.04 (1.03, 1.06)	3.7	1.02 (0.99, 1.05)
	4-5	16.4	25.5	1.09 (1.07, 1.10)	21.1	1.05 (1.03, 1.06)	4.6	1.22 (1.18, 1.27)
	6-9	32.3	24.3	1.10 (1.08, 1.12)	18.8	0.99 (0.97, 1.00)	5.9	1.59 (1.52, 1.65)
	10+	26.1	18.9	1.02 (1.00, 1.05)	12.9	0.83 (0.81, 0.85)	6.3	1.91 (1.80, 2.04)
Vaccination Claims[c]	No Claim	23.2	18.5	Ref	13.0	Ref	5.7	Ref
	1 or More	76.8	24.4	1.30 (1.27, 1.33)	19.6	1.46 (1.43, 1.49)	5.1	0.86 (0.82, 0.89)
Nirmatrelvir Contraindication[d]	None	10.8	23.3	Ref	20.3	Ref	3.2	Ref
	Relative	75.3	23.5	1.12 (1.10, 1.13)	18.7	1.05 (1.04, 1.06)	5.2	1.42 (1.38, 1.46)
	Strict	13.8	20.1	1.01 (0.99, 1.03)	13.2	0.81 (0.79, 0.83)	7.2	1.89 (1.80, 1.98)

© 2024 Wilcock AD et al. *JAMA Health Forum.*

[a] “Other” race includes American Indian or Alaska Native; Asian; Native Hawaiian or Pacific Islander; these are the four options for self-identified race in the Medicare enrollment file besides White and Black race or Hispanic/Latino. Less than 1% of beneficiaries are coded as “unknown” race.
[b] For the chronic condition count, the presence of 27 conditions was gathered from the Chronic Condition Data Warehouse, which uses claims since 1999 to describe Medicare beneficiaries’ accumulated chronic disease burden. Co-morbidities were defined as any condition present by as of December 31, 2020.
[c] Receipt of vaccination for COVID-19 over the period January 2020 through December 2022, classified as having ≥1 vaccine claims or having no vaccine claims, which could includes both unvaccinated beneficiaries and those who received vaccines without billing Medicare.
[d] We combined drug tiers and indicators for kidney or liver disease into 3 levels of potential nirmatrelvir contraindications: 1) no liver or kidney disease and no drug-drug interactions (no contraindication), 2) any tier 1 interactions and/or liver/kidney disease (moderate contraindication), and 3) any tier 2 drug interaction, with or without liver/kidney disease (severe contraindication).
[e] Telemedicine visits were identified through modifiers GT, GQ, or 95 on eligible outpatient services or CPT codes 99441-99443

© 2024 Wilcock AD et al. *JAMA Health Forum.*

eTable 3. Proportion of treatment doses proximate to a COVID-19 diagnosis

	Doses	% of Doses Proximate to a COVID-19 Diagnosis	
	N	+/- 10 Days	+/- 30 Days
**Oral Therapies**	1,069,331	59.5	62.1
Nirmatrelvir	938,671	58.2	61.0
Molnupiravir	131,281	68.4	70.3
**IV Therapies**	270,700	98.5	98.7
Monoclonal Antibodies	240,465	98.4	98.6
Remdesivir	30,457	99.6	99.7

Total doses were assessed in 2022, among Medicare beneficiaries with Parts A & B and no Medicare Advantage enrollment; Part D enrollment was not a requirement to reflect full use of Part B IV therapies.

© 2024 Wilcock AD et al. *JAMA Health Forum.*

eTable 4. Characteristics of oral therapy use with and without COVID-19 diagnosis

		Beneficiaries	Oral Fill with and without a COVID-19 diagnosis within +/- 10 days		% of Fills without a diagnosis
		N (Col %)	**With** Row %	**Without** Row %	
	Total, No.	20,026,910	3.1	2.1	40.4%
Age	Under 40	2.5	1.0	0.6	37.9%
	40-49	2.3	1.5	0.8	35.1%
	50-59	3.9	1.8	1.0	35.6%
	60-64	3.2	2.0	1.2	37.2%
	65-69	25.2	3.3	2.3	41.2%
	70-74	24.1	3.5	2.4	40.8%
	75-79	17.1	3.4	2.3	40.4%
	80-84	11.0	3.1	2.0	39.4%
	85-89	6.3	2.7	1.8	39.8%
	90+	4.3	2.3	1.6	40.8%
Sex	Female	56.8	3.1	2.1	39.7%
	Male	43.2	3.0	2.1	41.2%
Race	Asian	3.1	3.3	2.5	42.7%
	Black	6.5	1.7	0.8	32.0%
	Hispanic	5.2	2.4	1.2	32.6%
	White	81.5	3.2	2.2	40.7%
	Other Race[a]	3.7	3.3	2.6	44.0%
Geography	Urban	76.7	3.2	2.2	40.7%
	Rural	23.3	2.6	1.6	39.0%
Medicaid Eligibility	Non-Duals	78.0	3.5	2.4	41.1%
	Duals	22.0	1.7	0.9	34.8%
Original Reason for Medicare Eligibility	Age	80.2	3.3	2.3	41.0%
	Disability	19.2	2.0	1.1	36.2%
	ESRD	0.6	1.3	0.6	32.5%
Institution	Community	93.6	3.1	2.2	40.7%
	Nursing Home	6.4	2.0	1.0	32.6%
Chronic Condition Count[b]	No information	4.9	3.0	2.1	41.4%
	0	12.8	2.6	1.9	42.3%
	1-3	19.0	2.9	2.1	42.1%
	4-5	17.7	3.3	2.3	41.2%
	6-9	30.7	3.3	2.2	39.7%
	10+	19.8	2.9	1.7	37.6%
Vaccination Claims[c]	No Claim	26.0	2.0	1.1	35.7%
	1 or More	74.0	3.4	2.4	41.3%
Nirmatrelvir Contraindication[d]	None	16.5	2.3	1.6	41.7%
	Relative	72.4	3.3	2.2	40.3%
	Strict	11.2	2.8	1.8	38.9%
Telemedicine	No Use	26.0	2.7	2.0	43.2%
	Use	74.0	3.8	2.1	36.2%

[a] "Other" race includes American Indian or Alaska Native; Asian; Native Hawaiian or Pacific Islander; these are the four options for self-identified race in the Medicare enrollment file besides White and Black race or Hispanic/Latino. Less than 1% of beneficiaries are coded as "unknown" race.
[b] For the chronic condition count, the presence of 27 conditions was gathered from the Chronic Condition Data Warehouse, which uses claims since 1999 to describe Medicare beneficiaries' accumulated chronic disease burden. Co-morbidities were defined as any condition present by as of December 31, 2020.

© 2024 Wilcock AD et al. *JAMA Health Forum.*

[c] Receipt of vaccination for COVID-19 over the period January 2020 through December 2022, classified as having ≥1 vaccine claims or having no vaccine claims, which could includes both unvaccinated beneficiaries and those who received vaccines without billing Medicare.
[d] We combined drug tiers and indicators for kidney or liver disease into 3 levels of potential nirmatrelvir contraindications: 1) no liver or kidney disease and no drug-drug interactions (no contraindication), 2) any tier 1 interactions and/or liver/kidney disease (moderate contraindication), and 3) any tier 2 drug interaction, with or without liver/kidney disease (severe contraindication).

eTable 5. Rates of COVID-19 therapy by demographics in 2022

		Beneficiaries	Any COVID-19 Therapy		Oral COVID-19 Therapy		IV COVID-19 Therapy	
		N (Col %)	Row %	Adj. OR (95% CI)	Row %	Adj. OR (95% CI)	Row %	Adj. OR (95% CI)
	Total, No.	20,026,910	6.0	--	5.1	--	1.0	--
Age	Under 40	2.5	2.2	0.74 (0.71, 0.77)	1.7	0.70 (0.67, 0.72)	0.5	1.04 (0.94, 1.14)
	40-49	2.3	3.1	0.87 (0.85, 0.90)	2.3	0.85 (0.83, 0.88)	0.8	1.04 (0.96, 1.12)
	50-59	3.9	3.5	0.88 (0.86, 0.91)	2.8	0.89 (0.87, 0.91)	0.8	0.90 (0.85, 0.95)
	60-64	3.2	4.1	0.89 (0.87, 0.91)	3.2	0.89 (0.87, 0.91)	0.9	0.89 (0.85, 0.93)
	65-69	25.2	6.4	Ref	5.6	Ref	0.8	Ref
	70-74	24.1	6.8	0.93 (0.92, 0.94)	5.9	0.93 (0.93, 0.94)	1.0	0.91 (0.89, 0.93)
	75-79	17.1	6.7	0.85 (0.84, 0.86)	5.6	0.86 (0.84, 0.87)	1.1	0.86 (0.84, 0.88)
	80-84	11.0	6.2	0.77 (0.76, 0.79)	5.0	0.77 (0.75, 0.78)	1.2	0.83 (0.80, 0.85)
	85-89	6.3	5.7	0.71 (0.70, 0.73)	4.5	0.71 (0.69, 0.72)	1.2	0.79 (0.76, 0.82)
	90+	4.3	4.9	0.64 (0.62, 0.65)	3.8	0.63 (0.61, 0.65)	1.1	0.73 (0.70, 0.76)
Sex	Female	56.8	6.1	1.03 (1.02, 1.04)	5.2	1.04 (1.03, 1.05)	1.0	0.98 (0.97, 0.99)
	Male	43.2	6.0	Ref	5.0	Ref	1.0	Ref
Race	Asian	3.1	6.2	1.10 (1.01, 1.19)	5.7	1.19 (1.09, 1.31)	0.6	0.62 (0.54, 0.70)
	Black	6.5	3.0	0.56 (0.54, 0.58)	2.5	0.57 (0.55, 0.60)	0.6	0.52 (0.48, 0.57)
	Hispanic	5.2	4.3	0.86 (0.81, 0.92)	3.6	0.89 (0.83, 0.95)	0.7	0.76 (0.64, 0.90)
	White	81.5	6.4	Ref	5.4	Ref	1.0	Ref
	Other Race[a]	3.7	6.8	1.11 (1.09, 1.13)	5.9	1.12 (1.10, 1.15)	1.0	1.04 (0.99, 1.09)
Geography	Urban	76.7	6.3	Ref	5.4	Ref	1.0	Ref
	Rural	23.3	5.2	0.86 (0.83, 0.90)	4.2	0.82 (0.78, 0.85)	1.1	1.10 (0.96, 1.26)
Medicaid Eligibility	Non-Duals	78.0	6.8	Ref	5.8	Ref	1.0	Ref
	Duals	22.0	3.3	0.60 (0.57, 0.63)	2.6	0.58 (0.56, 0.61)	0.7	0.70 (0.62, 0.78)
Original Reason for Medicare Eligibility	Age	80.2	6.5	Ref	5.6	Ref	1.0	Ref
	Disability	19.2	4.0	0.82 (0.81, 0.83)	3.1	0.80 (0.78, 0.81)	0.9	0.95 (0.91, 0.99)
	ESRD	0.6	5.5	1.13 (1.06, 1.20)	2.0	0.48 (0.45, 0.51)	3.7	3.90 (3.62, 4.19)
Institution	Community	93.6	6.2	Ref	5.3	Ref	1.0	Ref
	Nursing Home	6.4	4.2	0.78 (0.75, 0.81)	3.0	0.71 (0.67, 0.75)	1.2	1.01 (0.96, 1.06)
Chronic Condition Count[b]	No information	4.9	5.7	1.20 (1.19, 1.22)	5.0	1.19 (1.18, 1.21)	0.7	1.32 (1.27, 1.38)
	0	12.8	5.0	Ref	4.5	Ref	0.6	Ref
	1-3	19.0	5.5	1.13 (1.11, 1.14)	4.9	1.13 (1.11, 1.14)	0.6	1.12 (1.08, 1.16)
	4-5	17.7	6.4	1.26 (1.24, 1.28)	5.6	1.23 (1.21, 1.25)	0.8	1.48 (1.42, 1.54)
	6-9	30.7	6.6	1.38 (1.35, 1.41)	5.5	1.28 (1.26, 1.31)	1.2	2.13 (2.05, 2.22)
	10+	19.8	6.1	1.47 (1.42, 1.51)	4.6	1.26 (1.22, 1.30)	1.6	2.99 (2.82, 3.17)
Vaccination Claims[c]	No Claim	26.0	4.0	Ref	3.1	Ref	1.0	Ref
	1 or More	74.0	6.7	1.44 (1.41, 1.48)	5.8	1.60 (1.56, 1.63)	1.0	0.88 (0.85, 0.93)
Nirmatrelvir Contraindication[d]	None	16.5	4.3	Ref	3.9	Ref	0.4	Ref
	Relative	72.4	6.4	1.41 (1.39, 1.42)	5.5	1.35 (1.34, 1.37)	1.0	1.87 (1.82, 1.93)
	Strict	11.2	6.1	1.36 (1.33, 1.39)	4.5	1.17 (1.14, 1.20)	1.7	2.69 (2.58, 2.81)

© 2024 Wilcock AD et al. *JAMA Health Forum.*

[a] "Other" race includes American Indian or Alaska Native; Asian; Native Hawaiian or Pacific Islander; these are the four options for self-identified race in the Medicare enrollment file besides White and Black race or Hispanic/Latino. Less than 1% of beneficiaries are coded as "unknown" race.
[b] For the chronic condition count, the presence of 27 conditions was gathered from the Chronic Condition Data Warehouse, which uses claims since 1999 to describe Medicare beneficiaries' accumulated chronic disease burden. Co-morbidities were defined as any condition present by as of December 31, 2020.
[c] Receipt of vaccination for COVID-19 over the period January 2020 through December 2022, classified as having ≥1 vaccine claims or having no vaccine claims, which could includes both unvaccinated beneficiaries and those who received vaccines without billing Medicare.
[d] We combined drug tiers and indicators for kidney or liver disease into 3 levels of potential nirmatrelvir contraindications: 1) no liver or kidney disease and no drug-drug interactions (no contraindication), 2) any tier 1 interactions and/or liver/kidney disease (moderate contraindication), and 3) any tier 2 drug interaction, with or without liver/kidney disease (severe contraindication).
[e] Telemedicine visits were identified through modifiers GT, GQ, or 95 on eligible outpatient services or CPT codes 99441-99443

© 2024 Wilcock AD et al. *JAMA Health Forum.*

eTable 6. Rates of COVID-19 therapy by demographics among the unvaccinated in 2022

		Beneficiaries	Any COVID-19 Therapy		Oral COVID-19 Therapy		IV COVID-19 Therapy	
		N (col %)	Row %	Adj. OR (95% CI)	Row %	Adj. OR (95% CI)	Row %	Adj. OR (95% CI)
	Total, No.	5,216,134	4.0	--	3.1	--	1.0	--
Age	Under 40	5.4	1.5	0.76 (0.72, 0.79)	1.1	0.73 (0.7, 0.77)	0.4	0.87 (0.78, 0.97)
	40-49	4.4	2.2	0.88 (0.84, 0.92)	1.7	0.88 (0.84, 0.92)	0.6	0.89 (0.82, 0.97)
	50-59	6.7	2.6	0.88 (0.85, 0.91)	2.0	0.91 (0.87, 0.94)	0.7	0.82 (0.76, 0.88)
	60-64	5.0	3.1	0.89 (0.86, 0.93)	2.4	0.92 (0.89, 0.95)	0.7	0.83 (0.78, 0.88)
	65-69	27.1	4.3	Ref	3.5	Ref	0.9	Ref
	70-74	20.5	4.7	0.93 (0.91, 0.94)	3.7	0.92 (0.91, 0.94)	1.1	0.94 (0.91, 0.98)
	75-79	13.5	4.8	0.85 (0.84, 0.87)	3.6	0.85 (0.83, 0.88)	1.2	0.87 (0.84, 0.91)
	80-84	8.7	4.6	0.79 (0.78, 0.81)	3.4	0.78 (0.76, 0.8)	1.3	0.86 (0.82, 0.9)
	85-89	5.1	4.4	0.75 (0.72, 0.77)	3.2	0.73 (0.7, 0.75)	1.3	0.82 (0.78, 0.87)
	90+	3.7	3.9	0.65 (0.63, 0.68)	2.8	0.64 (0.61, 0.66)	1.2	0.73 (0.68, 0.79)
Sex	Female	54.9	4.3	1.16 (1.15, 1.17)	3.4	1.18 (1.16, 1.19)	1.0	1.1 (1.08, 1.12)
	Male	45.1	3.7	Ref	2.8	Ref	0.9	Ref
Race	Asian	2.4	3.7	1.06 (0.94, 1.2)	3.2	1.23 (1.07, 1.42)	0.5	0.55 (0.47, 0.65)
	Black	8.9	1.9	0.56 (0.52, 0.59)	1.5	0.59 (0.56, 0.62)	0.4	0.47 (0.42, 0.53)
	Hispanic	7.8	2.8	0.84 (0.76, 0.93)	2.3	0.91 (0.82, 1)	0.6	0.65 (0.53, 0.81)
	White	77.4	4.4	Ref	3.4	Ref	1.1	Ref
	Other Race[a]	3.5	3.8	1.03 (1, 1.07)	2.9	1.04 (0.99, 1.08)	0.9	1.03 (0.95, 1.11)
Geography	Urban	68.2	4.0	Ref	3.2	Ref	0.9	Ref
	Rural	31.8	4.1	0.95 (0.91, 1)	3.0	0.9 (0.86, 0.94)	1.1	1.14 (1, 1.31)
Medicaid Eligibility	Non-Duals	64.7	4.9	Ref	3.9	Ref	1.1	Ref
	Duals	35.3	2.4	0.58 (0.54, 0.61)	1.8	0.55 (0.53, 0.57)	0.6	0.69 (0.59, 0.8)
Original Reason for Medicare Eligibility	Age	69.8	4.6	Ref	3.6	Ref	1.0	Ref
	Disability	29.5	2.8	0.82 (0.79, 0.84)	2.1	0.8 (0.78, 0.82)	0.7	0.87 (0.81, 0.94)
	ESRD	0.7	4.2	1.19 (1.1, 1.28)	1.4	0.5 (0.45, 0.55)	2.9	3.52 (3.21, 3.85)
Institution	Community	94.4	4.1	Ref	3.2	Ref	0.9	Ref
	Nursing Home	5.6	3.8	0.91 (0.88, 0.95)	2.5	0.83 (0.79, 0.87)	1.3	1.11 (1.05, 1.18)
Chronic Condition Count[b]	No information	8.2	3.9	1.36 (1.32, 1.4)	3.2	1.35 (1.31, 1.39)	0.7	1.37 (1.29, 1.46)
	0	19.7	3.1	Ref	2.5	Ref	0.5	Ref
	1-3	21.1	3.2	1.18 (1.15, 1.21)	2.6	1.17 (1.14, 1.21)	0.6	1.2 (1.14, 1.27)
	4-5	16.3	4.2	1.39 (1.35, 1.44)	3.4	1.35 (1.31, 1.4)	0.9	1.56 (1.47, 1.65)
	6-9	26.2	4.8	1.6 (1.55, 1.65)	3.7	1.49 (1.44, 1.54)	1.2	2.1 (1.99, 2.21)
	10+	16.8	4.9	1.77 (1.69, 1.85)	3.4	1.53 (1.46, 1.6)	1.6	2.75 (2.57, 2.94)
Nirmatrelvir Contraindication[d]	None	23.6	2.3	Ref	1.9	Ref	0.4	Ref
	Relative	66.0	4.5	1.8 (1.76, 1.83)	3.5	1.76 (1.73, 1.8)	1.1	1.94 (1.85, 2.02)
	Strict	10.4	4.8	1.88 (1.83, 1.93)	3.3	1.65 (1.6, 1.71)	1.6	2.63 (2.5, 2.77)

[a] "Other" race includes American Indian or Alaska Native; Asian; Native Hawaiian or Pacific Islander; these are the four options for self-identified race in the Medicare enrollment file besides White and Black race or Hispanic/Latino. Less than 1% of beneficiaries are coded as "unknown" race.

© 2024 Wilcock AD et al. *JAMA Health Forum.*

[b] For the chronic condition count, the presence of 27 conditions was gathered from the Chronic Condition Data Warehouse, which uses claims since 1999 to describe Medicare beneficiaries' accumulated chronic disease burden. Co-morbidities were defined as any condition present by as of December 31, 2020.
[c] Receipt of vaccination for COVID-19 over the period January 2020 through December 2022, classified as having ≥1 vaccine claims or having no vaccine claims, which could includes both unvaccinated beneficiaries and those who received vaccines without billing Medicare.
[d] We combined drug tiers and indicators for kidney or liver disease into 3 levels of potential nirmatrelvir contraindications: 1) no liver or kidney disease and no drug-drug interactions (no contraindication), 2) any tier 1 interactions and/or liver/kidney disease (moderate contraindication), and 3) any tier 2 drug interaction, with or without liver/kidney disease (severe contraindication).
[e] Telemedicine visits were identified through modifiers GT, GQ, or 95 on eligible outpatient services or CPT codes 99441-99443

© 2024 Wilcock AD et al. *JAMA Health Forum.*

eTable 7. Rates of COVID-19 therapy by demographics among the aged 65 years and over in 2022

		Beneficiaries	Any COVID-19 Therapy		Oral COVID-19 Therapy		IV COVID-19 Therapy	
		N (Col %)	Row %	Adj. OR (95% CI)	Row %	Adj. OR (95% CI)	Row %	Adj. OR (95% CI)
	Total, No.	20,026,910	6.0	--	5.1	--	6.0	--
Age	Under 40	--	--	--	--	--	--	--
	40-49	--	--	--	--	--	--	--
	50-59	--	--	--	--	--	--	--
	60-64	--	--	--	--	--	--	--
	65-69	28.6	6.4	Ref	5.6	Ref	0.8	Ref
	70-74	27.4	6.8	0.93 (0.93, 0.94)	5.9	0.94 (0.93, 0.95)	1.0	0.90 (0.89, 0.92)
	75-79	19.4	6.7	0.86 (0.85, 0.87)	5.6	0.86 (0.85, 0.88)	1.1	0.85 (0.84, 0.87)
	80-84	12.5	6.2	0.78 (0.77, 0.80)	5.0	0.78 (0.76, 0.79)	1.2	0.82 (0.80, 0.85)
	85-89	7.2	5.7	0.73 (0.71, 0.74)	4.5	0.72 (0.70, 0.73)	1.2	0.79 (0.76, 0.81)
	90+	4.9	4.9	0.65 (0.63, 0.67)	3.8	0.64 (0.62, 0.66)	1.1	0.73 (0.69, 0.76)
Sex	Female	58.0	6.3	1.00 (0.99, 1.01)	5.4	1.01 (1.00, 1.02)	1.0	0.93 (0.92, 0.95)
	Male	42.0	6.6	Ref	5.5	Ref	1.1	Ref
Race	Asian	3.2	6.5	1.11 (1.03, 1.21)	6.0	1.21 (1.11, 1.33)	0.6	0.60 (0.52, 0.68)
	Black	5.1	3.4	0.55 (0.53, 0.57)	2.8	0.56 (0.54, 0.59)	0.6	0.50 (0.45, 0.55)
	Hispanic	4.5	4.6	0.85 (0.80, 0.92)	3.9	0.88 (0.82, 0.94)	0.7	0.75 (0.62, 0.90)
	White	83.6	6.6	Ref	5.6	Ref	1.1	Ref
	Other Race[a]	3.6	7.5	1.12 (1.10, 1.14)	6.5	1.13 (1.11, 1.15)	1.0	1.028 (0.98, 1.08)
Geography	Urban	77.1	6.7	Ref	5.7	Ref	1.0	Ref
	Rural	22.9	5.5	0.85 (0.81, 0.89)	4.5	0.81 (0.78, 0.85)	1.1	1.09 (0.95, 1.25)
Medicaid Eligibility	Non-Duals	85.6	6.9	Ref	5.9	Ref	1.0	Ref
	Duals	14.4	3.6	0.60 (0.56, 0.63)	2.8	0.58 (0.55, 0.61)	0.8	0.72 (0.63, 0.82)
Original Reason for Medicare Eligibility	Age	90.8	6.5	Ref	5.6	Ref	1.0	Ref
	Disability	9.0	5.1	0.83 (0.82, 0.85)	4.0	0.81 (0.79, 0.82)	1.2	0.94 (0.90, 0.98)
	ESRD	0.2	7.0	1.07 (1.00, 1.14)	2.7	0.50 (0.46, 0.53)	4.5	3.63 (3.31, 3.99)
Institution	Community	93.4	6.6	Ref	5.6	Ref	1.0	Ref
	Nursing Home	6.6	4.3	0.78 (0.75, 0.81)	3.1	0.71 (0.67, 0.75)	1.2	1.01 (0.96, 1.06)
Chronic Condition Count[b]	No information	4.4	6.2	1.16 (1.15, 1.18)	5.5	1.16 (1.15, 1.18)	0.7	1.21 (1.15, 1.26)
	0	11.9	5.5	Ref	5.0	Ref	0.6	Ref
	1-3	17.9	6.1	1.12 (1.11, 1.14)	5.6	1.12 (1.11, 1.14)	0.6	1.11 (1.07, 1.15)
	4-5	17.7	6.8	1.24 (1.22, 1.26)	6.0	1.21 (1.19, 1.23)	0.8	1.46 (1.40, 1.51)
	6-9	31.5	6.9	1.35 (1.33, 1.38)	5.8	1.26 (1.24, 1.29)	1.2	2.11 (2.02, 2.20)
	10+	21.0	6.2	1.43 (1.38, 1.48)	4.7	1.23 (1.20, 1.27)	1.6	2.96 (2.78, 3.15)
Vaccination Claims[c]	No Claim	23.2	4.5	Ref	3.5	Ref	1.1	Ref
	1 or More	76.8	7.0	1.43 (1.39, 1.46)	6.0	1.59 (1.55, 1.63)	1.0	0.85 (0.81, 0.89)
Nirmatrelvir Contraindication[d]	None	16.0	4.7	Ref	4.3	Ref	0.4	Ref
	Relative	72.9	6.8	1.38 (1.363, 1.398)	5.8	1.33 (1.32, 1.35)	1.0	1.80 (1.75, 1.85)
	Strict	11.1	6.5	1.34 (1.31, 1.37)	4.8	1.15 (1.12, 1.19)	1.7	2.63 (2.52, 2.75)

© 2024 Wilcock AD et al. *JAMA Health Forum.*

[a] “Other” race includes American Indian or Alaska Native; Asian; Native Hawaiian or Pacific Islander; these are the four options for self-identified race in the Medicare enrollment file besides White and Black race or Hispanic/Latino. Less than 1% of beneficiaries are coded as “unknown” race.
[b] For the chronic condition count, the presence of 27 conditions was gathered from the Chronic Condition Data Warehouse, which uses claims since 1999 to describe Medicare beneficiaries’ accumulated chronic disease burden. Co-morbidities were defined as any condition present by as of December 31, 2020.
[c] Receipt of vaccination for COVID-19 over the period January 2020 through December 2022, classified as having ≥1 vaccine claims or having no vaccine claims, which could includes both unvaccinated beneficiaries and those who received vaccines without billing Medicare.
[d] We combined drug tiers and indicators for kidney or liver disease into 3 levels of potential nirmatrelvir contraindications: 1) no liver or kidney disease and no drug-drug interactions (no contraindication), 2) any tier 1 interactions and/or liver/kidney disease (moderate contraindication), and 3) any tier 2 drug interaction, with or without liver/kidney disease (severe contraindication).
[e] Telemedicine visits were identified through modifiers GT, GQ, or 95 on eligible outpatient services or CPT codes 99441-99443

© 2024 Wilcock AD et al. *JAMA Health Forum.*

eTable 8. Rates of oral COVID-19 therapy by basic demographics among Medicare Advantage and traditional Medicare in 2022

		Traditional Medicare			Medicare Advantage		
		Beneficiaries	Oral COVID-19 Therapy		Beneficiaries	Oral COVID-19 Therapy	
		N (Col %)	Row %	Adj. OR (95% CI)	N (Col %)	Row %	Adj. OR (95% CI)
	Total, No.	20,026,910	5.1	--	27,306,324	3.5	--
Age	Under 40	2.5	1.7	0.58 (0.55, 0.6)	1.3	1.4	0.72 (0.68, 0.76)
	40-49	2.3	2.3	0.79 (0.76, 0.81)	1.9	1.9	0.89 (0.85, 0.93)
	50-59	3.9	2.8	0.87 (0.85, 0.89)	5.2	2.2	0.94 (0.91, 0.96)
	60-64	3.2	3.2	0.89 (0.87, 0.91)	5.3	2.5	0.98 (0.96, 0.99)
	65-69	25.2	5.6	Ref	26.4	3.4	Ref
	70-74	24.1	5.9	1.02 (1.01, 1.03)	23.9	3.9	1.09 (1.08, 1.1)
	75-79	17.1	5.6	0.98 (0.96, 0.99)	16.9	4.0	1.11 (1.09, 1.13)
	80-84	11.0	5.0	0.89 (0.87, 0.91)	10.3	3.8	1.07 (1.05, 1.09)
	85-89	6.3	4.5	0.82 (0.8, 0.84)	5.6	3.6	1.01 (0.98, 1.03)
	90+	4.3	3.8	0.73 (0.71, 0.75)	3.2	3.3	0.94 (0.91, 0.97)
Sex	Female	56.8	5.2	1.03 (1.03, 1.04)	56.7	3.6	1.11 (1.1, 1.12)
	Male	43.2	5.0	Ref	43.3	3.4	Ref
Race	Asian	3.1	5.7	1.22 (1.11, 1.35)	4.2	4.3	1.21 (1.09, 1.34)
	Black	6.5	2.5	0.57 (0.55, 0.6)	13.4	1.8	0.55 (0.52, 0.58)
	Hispanic	5.2	3.6	0.86 (0.8, 0.92)	11.5	2.9	0.87 (0.79, 0.95)
	White	81.5	5.4	Ref	68.2	3.9	Ref
	Other Race[a]	3.7	5.9	1.14 (1.11, 1.16)	2.7	4.0	1.07 (1.03, 1.1)
Geography	Urban	76.7	5.4	Ref	83.5	3.6	Ref
	Rural	23.3	4.2	0.77 (0.74, 0.81)	16.5	3.1	0.85 (0.8, 0.89)
Medicaid Eligibility	Non-Duals	78.0	5.8	Ref	75.2	4.0	Ref
	Duals	22.0	2.6	0.55 (0.53, 0.57)	24.8	1.9	0.54 (0.5, 0.57)
OREC	Age	80.2	5.6	Ref	75.5	3.8	Ref
	Disability	19.2	3.1	0.84 (0.83, 0.86)	24.2	2.5	0.89 (0.87, 0.91)
	ESRD	0.6	2.0	0.56 (0.53, 0.6)	0.3	1.3	0.54 (0.49, 0.59)
Institutionalization	Community	93.9	5.3	Ref	95.6	3.5	Ref
	Nursing Home	6.1	2.9	0.74 (0.7, 0.78)	4.4	2.7	0.89 (0.86, 0.92)

[a] "Other" race includes American Indian or Alaska Native; Asian; Native Hawaiian or Pacific Islander; these are the four options for self-identified race in the Medicare enrollment file besides White and Black race or Hispanic/Latino. Less than 1% of beneficiaries are coded as "unknown" race.

© 2024 Wilcock AD et al. *JAMA Health Forum.*

eTable 9. Rates of COVID-19 therapies and other services by severity risk quintile

	Beneficiaries N (Col %)	Any COVID-19 Therapy Row %	Oral COVID-19 Therapy Row %	IV COVID-19 Therapy Row %	COVID-19 Test Row %	COVID-19 E&M Visit Row %	COVID-19 Inpatient Row %
Full study population							
Total, No.	20,026,910	6.0	5.1	1.0	32.9	10.7	1.0
Risk Quintiles							
Lowest	20.0	7.6	6.9	0.8	34.3	10.0	0.2
Low-mid	20.0	6.7	5.9	0.8	31.8	9.4	0.3
Middle	20.0	5.8	4.9	0.9	31.2	9.6	0.6
Mid-high	20.0	5.2	4.2	1.1	31.9	10.4	1.1
Highest	20.0	4.9	3.7	1.3	35.4	14.1	2.6
Among COVID-19 patients							
Total, No.	3,715,664	23.0	18.1	5.2	59.4	57.8	5.1
Risk Quintiles							
Lowest	20.0	26.9	23.3	3.9	57.9	51.9	0.9
Low-mid	20.0	26.4	22.0	4.6	58.0	54.0	1.7
Middle	20.0	24.1	18.8	5.6	60.9	57.7	3.6
Mid-high	20.0	21.4	15.4	6.3	61.9	60.9	7.0
Highest	20.0	16.4	10.9	5.7	58.4	64.3	12.5

See eMethods 2 for details on construction of the COVID-19 severity risk score for beneficiaries.

© 2024 Wilcock AD et al. *JAMA Health Forum.*

eTable 10. Impact of further adjustment for hospital region or primary care practice on adjusted effect sizes

		Sample Average	Unadjusted Effect	Control Variables	Control Variables + Hospital Referral Region		Control Variables + Primary Care Practice	
		N (Col %)	Coef. (95% CI)	Coef. (95% CI)	Coef. (95% CI)	% Chg	Coef. (95% CI)	% Chg
	Total, No.	20,026,910						
Age	Under 40	2.2	-4.24 (-4.41, -4.07)	-0.23 (-0.36, -0.11)	-0.17 (-0.31, -0.02)	-29.5	-0.29 (-0.39, -0.19)	22.6
	40-49	3.1	-3.35 (-3.50, -3.20)	-0.21 (-0.33, -0.08)	-0.16 (-0.3, -0.02)	-21.6	-0.20 (-0.30, -0.09)	-5.3
	50-59	3.5	-2.87 (-3.02, -2.72)	-0.41 (-0.52, -0.29)	-0.40 (-0.52, -0.28)	-1.2	-0.37 (-0.46, -0.27)	-9.1
	60-64	4.1	-2.35 (-2.51, -2.20)	-0.50 (-0.60, -0.40)	-0.51 (-0.61, -0.42)	2.4	-0.39 (-0.47, -0.30)	-22.2
	65-69 (Reference)	6.4						
	70-74	6.8	0.42 (0.35, 0.48)	-0.33 (-0.38, -0.28)	-0.35 (-0.39, -0.30)	3.9	-0.32 (-0.36, -0.28)	-3.9
	75-79	6.7	0.24 (0.14, 0.35)	-0.83 (-0.91, -0.75)	-0.87 (-0.94, -0.80)	4.6	-0.84 (-0.91, -0.77)	1.0
	80-84	6.2	-0.25 (-0.37, -0.12)	-1.42 (-1.52, -1.31)	-1.46 (-1.55, -1.37)	2.8	-1.41 (-1.50, -1.32)	-0.7
	85-89	5.7	-0.73 (-0.87, -0.59)	-1.84 (-1.96, -1.73)	-1.91 (-2.01, -1.81)	3.8	-1.82 (-1.92, -1.73)	-1.1
	90+	4.9	-1.47 (-1.64, -1.30)	-2.40 (-2.56, -2.25)	-2.49 (-2.64, -2.35)	3.7	-2.37 (-2.50, -2.23)	-1.3
Sex	Female	6.1	0.07 (0.03, 0.12)	0.11 (0.07, 0.15)	0.10 (0.07, 0.14)	-7.1	0.05 (0.01, 0.09)	-57.7
	Male (Reference)	6.0						
Race	Asian	6.2	-0.14 (-0.60, 0.33)	0.40 (-0.06, 0.86)	-0.01 (-0.29, 0.27)	-102.5	0.05 (-0.15, 0.26)	-86.7
	Black	3.0	-3.37 (-3.57, -3.17)	-2.25 (-2.43, -2.07)	-2.43 (-2.62, -2.23)	8.0	-2.04 (-2.16, -1.92)	-9.3
	Hispanic	4.3	-2.09 (-2.43, -1.76)	-0.74 (-1.02, -0.46)	-1.04 (-1.36, -0.72)	40.2	-0.58 (-0.74, -0.43)	-21.4
	White (Reference)	6.4						
	Other Race[a]	6.8	0.47 (0.32, 0.62)	0.63 (0.51, 0.76)	0.51 (0.39, 0.62)	-20.2	0.41 (0.33, 0.50)	-34.7
Geography	Urban (Reference)	6.3						
	Rural	5.2	-1.06 (-1.31, -0.81)	-0.83 (-1.07, -0.58)	-0.62 (-0.76, -0.49)	-24.5	-0.38 (-0.48, -0.28)	-54.0
Medicaid Eligibility	Non-Duals (Reference)	6.8						
	Duals	3.3	-3.56 (-3.73, -3.39)	-2.28 (-2.45, -2.12)	-2.42 (-2.62, -2.21)	6.1	-1.93 (-2.06, -1.80)	-15.4
Original Reason for Medicare Eligibility	Age (Reference)	6.5						
	Disability	4.0	-2.57 (-2.72, -2.43)	-1.11 (-1.21, -1.01)	-1.08 (-1.15, -1.00)	-2.7	-0.91 (-0.97, -0.84)	-18.1
	ESRD	5.5	-1.03 (-1.40, -0.66)	0.05 (-0.28, 0.39)	0.14 (-0.20, 0.47)	162.0	0.12 (-0.20, 0.44)	137.0
Institution	Community (Reference)	6.2						
	Nursing Home	4.2	-2.02 (-2.23, -1.81)	-1.24 (-1.42, -1.07)	-1.22 (-1.37, -1.07)	-1.6	-0.95 (-1.08, -0.83)	-23.1
Chronic Condition Count[b]	No information	5.7	1.11 (1.04, 1.19)	0.85 (0.78, 0.92)	0.86 (0.80, 0.93)	1.5	1.03 (0.97, 1.10)	21.5
	0 (Reference)	5.0						
	1-3	5.5	0.90 (0.83, 0.96)	0.50 (0.44, 0.55)	0.52 (0.47, 0.57)	5.2	0.20 (0.14, 0.25)	-60.7
	4-5	6.4	1.78 (1.70, 1.86)	1.09 (1.03, 1.16)	1.11 (1.05, 1.17)	1.8	0.72 (0.66, 0.79)	-33.7
	6-9	6.6	2.04 (1.93, 2.15)	1.62 (1.53, 1.71)	1.61 (1.53, 1.69)	-0.6	1.19 (1.12, 1.27)	-26.5
	10+	6.1	1.51 (1.31, 1.70)	1.92 (1.74, 2.10)	1.83 (1.71, 1.96)	-4.7	1.42 (1.32, 1.52)	-26.0
Vaccination Claims[c]	No Claim (Reference)	4.0						
	1 or More	6.7	2.71 (2.53, 2.88)	1.76 (1.64, 1.87)	1.67 (1.59, 1.76)	-5.1	1.37 (1.30, 1.44)	-22.2
Nirmatrelvir Contraindication[d]	None (Reference)	4.3						
	Relative	6.4	2.16 (2.06, 2.25)	1.64 (1.57, 1.71)	1.62 (1.54, 1.69)	-1.2	1.43 (1.36, 1.5)	-12.8

© 2024 Wilcock AD et al. *JAMA Health Forum.*

Strict	6.1	1.81 (1.66, 1.95)	1.44 (1.34, 1.53)	1.44 (1.34, 1.54)	0.0	1.26 (1.17, 1.36)	-12.5
Model R2	--	--	0.008	0.01	25.0	0.021	110.0

[a] "Other" race includes American Indian or Alaska Native; Asian; Native Hawaiian or Pacific Islander; these are the four options for self-identified race in the Medicare enrollment file besides White and Black race or Hispanic/Latino. Less than 1% of beneficiaries are coded as "unknown" race.
[b] For the chronic condition count, the presence of 27 conditions was gathered from the Chronic Condition Data Warehouse, which uses claims since 1999 to describe Medicare beneficiaries' accumulated chronic disease burden. Co-morbidities were defined as any condition present by as of December 31, 2020.
[c] Receipt of vaccination for COVID-19 over the period January 2020 through December 2022, classified as having ≥1 vaccine claims or having no vaccine claims, which could includes both unvaccinated beneficiaries and those who received vaccines without billing Medicare.
[d] We combined drug tiers and indicators for kidney or liver disease into 3 levels of potential nirmatrelvir contraindications: 1) no liver or kidney disease and no drug-drug interactions (no contraindication), 2) any tier 1 interactions and/or liver/kidney disease (moderate contraindication), and 3) any tier 2 drug interaction, with or without liver/kidney disease (severe contraindication).

© 2024 Wilcock AD et al. *JAMA Health Forum.*

eFigure 1. Hospitalizations and mortality prevent with counterfactual redistribution of nirmatrelvir in 2022

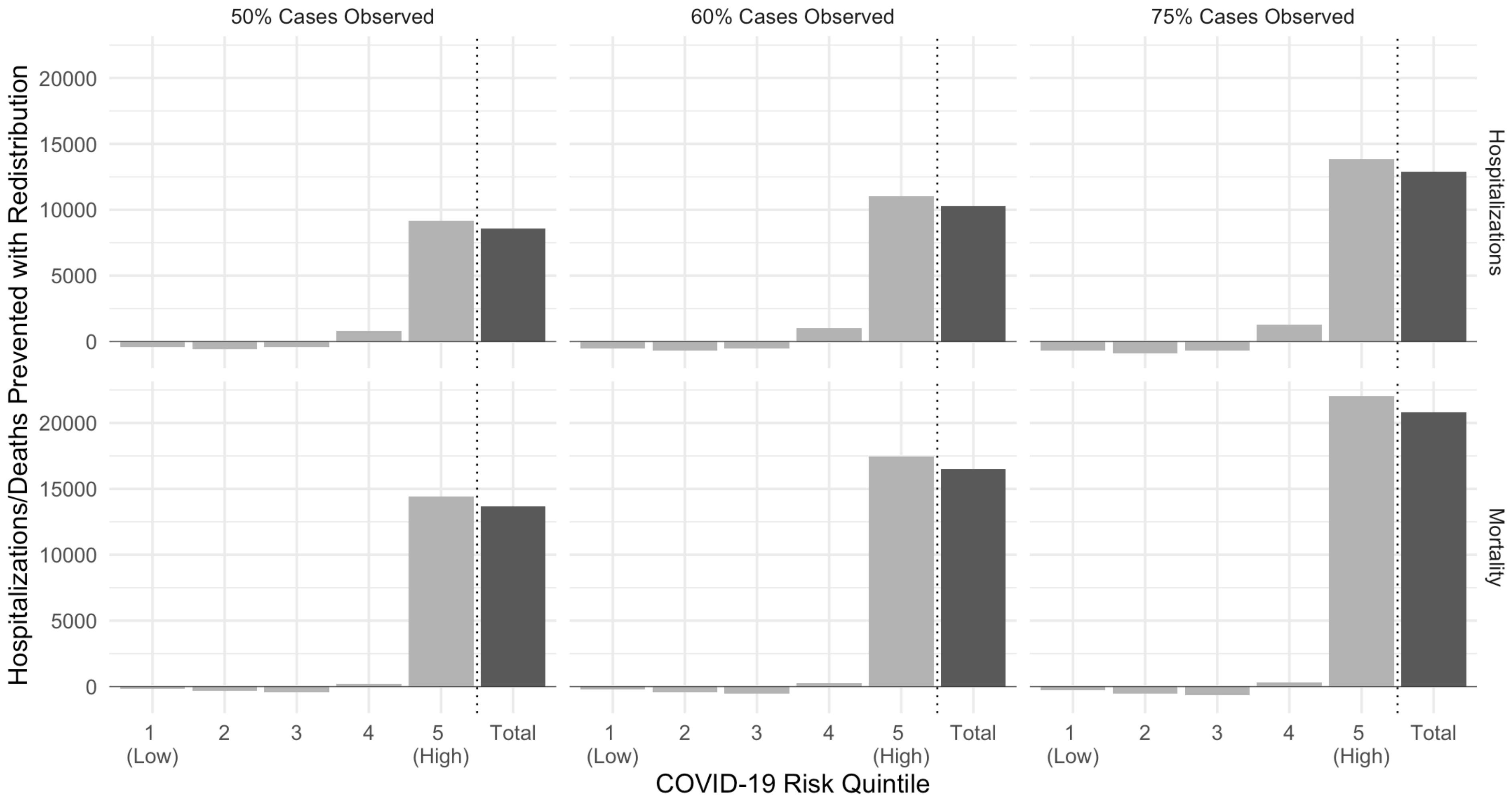

See eMethods 4 for details. We assumed a σ = 40% reduction in the risk of hospitalization and a σ = 70% reduction in mortality risk. Using these values, we calculated the counterfactual change in adverse outcomes (hospitalizations or mortality) under the treatment redistribution scenarios outlined above, under a range of case ascertainment rates *a* from 50% to 75%. As a lower bound, assuming 50% ascertainment and 50% treatment efficacy, reallocation of nirmatrelvir would have averted an estimated 9,671 deaths (9.74% reduction); and as an upper bound, assuming 75% ascertainment and 90% treatment efficacy, reallocation would have averted an estimated 27,135 deaths (27.3% reduction).

© 2024 Wilcock AD et al. *JAMA Health Forum.*
