# Supplementary material for: Clinical Risk and Outpatient Therapy Utilization for COVID-19 in the Medicare Population
Source: JAMA Health Forum. 2024 Jan 26;5(1):e235044. doi: 10.1001/jamahealthforum.2023.5044 (PMC10818223; doi:10.1001/jamahealthforum.2023.5044)
Supplement: Supplement 2. — Data Sharing Statement [file jamahealthforum-e235044-s002.pdf]

Data Sharing Statement

Wilcock. Clinical Risk and Outpatient Therapy Utilization for COVID-19 in the Medicare Population. *JAMA Health Forum*. Published January 26, 2024.
doi:10.1001/jamahealthforum.2023.5044

Data
Data available: No
